# Supplementary material for: Impact of excessive social media use on adolescent depression and its consequences in France: An individual-based microsimulation model
Source: PLoS Med. 2025 Oct 21;22(10):e1004737. doi: 10.1371/journal.pmed.1004737 (PMC12539716; doi:10.1371/journal.pmed.1004737)
Supplement: S1 Fig — (DOCX) [file pmed.1004737.s001.docx]

# S1 Fig. General Shape of Social Media Adoption Curve.

Note: Data used to construct the curve is based on regular use data between 2017 and 2020 [1].

# References

1. Asselin C. Les réseaux sociaux en France et dans le monde : les chiffres d’utilisation en 2021. In: DIGIMIND. Médiamétrie et Médiamétrie/NetRatings. [Internet]. 21 Apr 2021 [cited 25 Aug 2023]. Available: https://blog.digimind.com/fr/tendances/r%C3%A9seaux-sociaux-france-monde-chiffres-utilisation-2021
