# Supplementary material for: Impact of excessive social media use on adolescent depression and its consequences in France: An individual-based microsimulation model
Source: PLoS Med. 2025 Oct 21;22(10):e1004737. doi: 10.1371/journal.pmed.1004737 (PMC12539716; doi:10.1371/journal.pmed.1004737)
Supplement: S2 Fig — (DOCX) [file pmed.1004737.s002.docx]

# S2 Fig. Sex-stratified Social Media Adoption Curve Assumption.


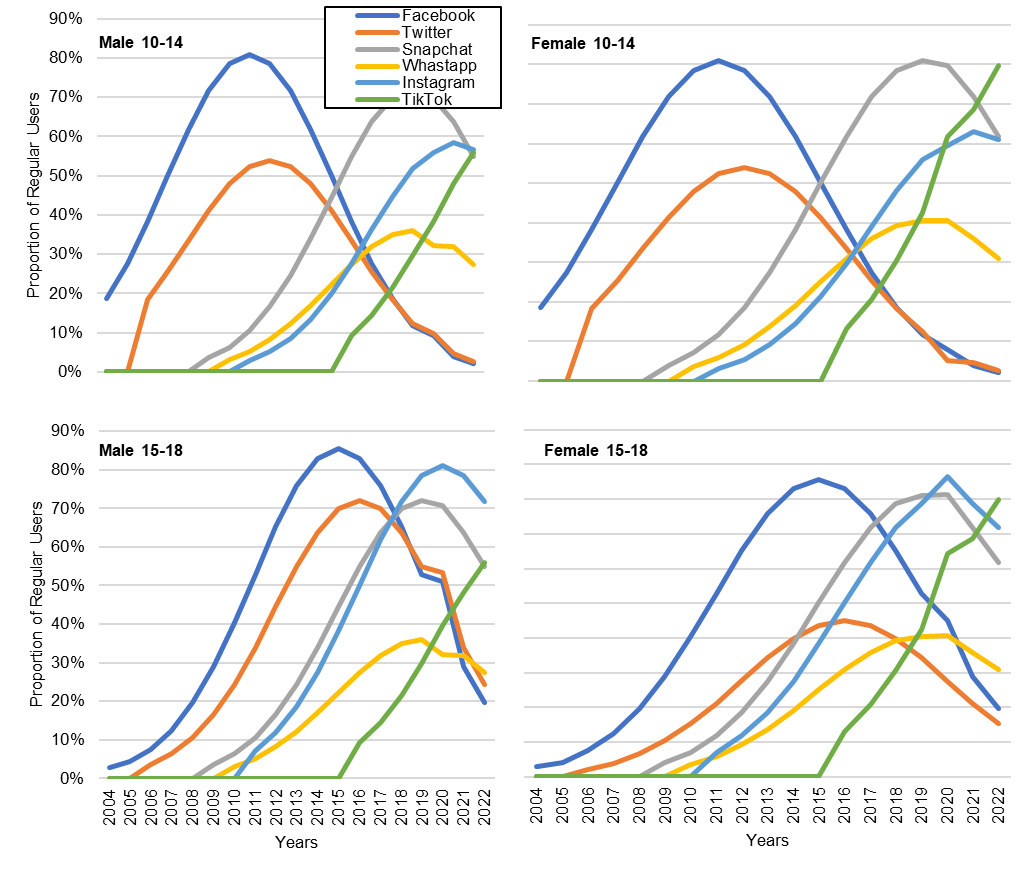


*Note: The curves are derived from the Bass diffusion model applied to historical data from Mediamétrie, accounting for each platform's market entry year (Facebook 2004, Twitter 2006, WhatsApp 2009, Instagram 2010, Snapchat 2011, TikTok 2016) and typical adolescent adoption patterns. The S-shaped curves reflect initial slow adoption, rapid growth phase, and eventual market saturation dynamics observed in real-world social media uptake among French adolescents.*
