# Supplementary material for: Impact of excessive social media use on adolescent depression and its consequences in France: An individual-based microsimulation model
Source: PLoS Med. 2025 Oct 21;22(10):e1004737. doi: 10.1371/journal.pmed.1004737 (PMC12539716; doi:10.1371/journal.pmed.1004737)
Supplement: S3 Fig — (DOCX) [file pmed.1004737.s003.docx]

# S3 Fig. Projected distribution of the individual risk of depression in the simulated population using or not social media, and projected effect of using social media more than 6 hours per day on this risk.

Individuals with an individual risk above 0.5 had depression in the simulation model.
