# Supplementary material for: Impact of excessive social media use on adolescent depression and its consequences in France: An individual-based microsimulation model
Source: PLoS Med. 2025 Oct 21;22(10):e1004737. doi: 10.1371/journal.pmed.1004737 (PMC12539716; doi:10.1371/journal.pmed.1004737)
Supplement: S4 Fig — (DOCX) [file pmed.1004737.s004.docx]

# S4 Fig. Average (95% CI) Distribution of Time Spent on Social Media by Adolescents Simulated with the Model compared to observed data in France.


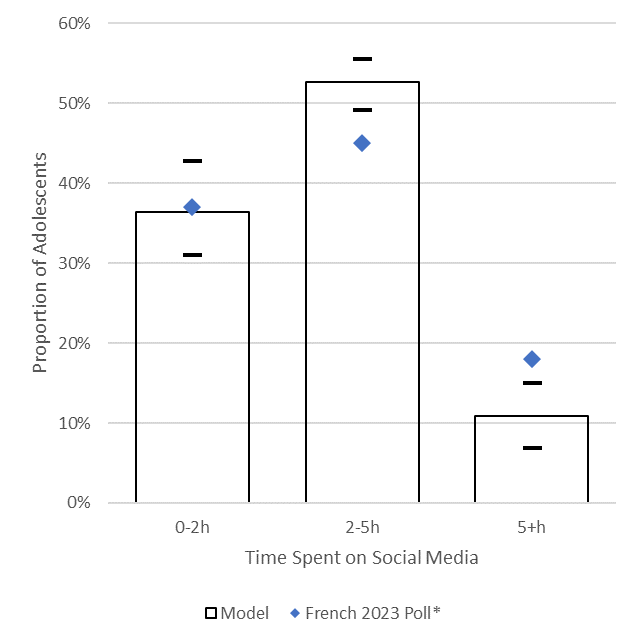


Note: The model included 2,312 people aged 16-25 years between March and April 2023 [2]; R² = 0.99 ; Pearson’s R = 0.99.
