# Supplementary material for: Impact of excessive social media use on adolescent depression and its consequences in France: An individual-based microsimulation model
Source: PLoS Med. 2025 Oct 21;22(10):e1004737. doi: 10.1371/journal.pmed.1004737 (PMC12539716; doi:10.1371/journal.pmed.1004737)
Supplement: S5 Fig — (DOCX) [file pmed.1004737.s005.docx]

# S5 Fig. Residual plots and diagnostic plots for model calibration and validation showing prediction accuracy and model assumptions.


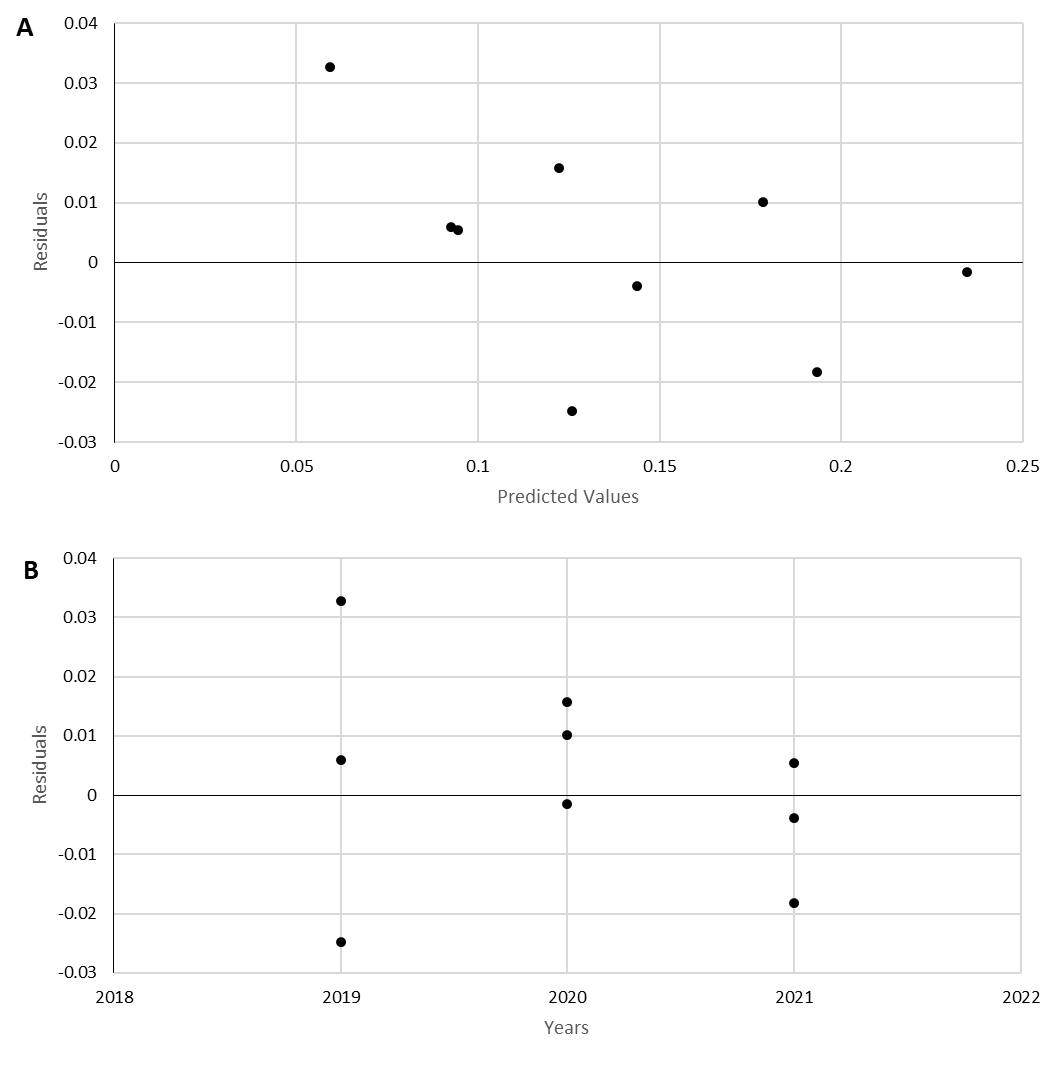


Note: Residual analysis showed no systematic bias patterns, and diagnostic plots confirmed adequate model assumptions.
