# Supplementary material for: Impact of excessive social media use on adolescent depression and its consequences in France: An individual-based microsimulation model
Source: PLoS Med. 2025 Oct 21;22(10):e1004737. doi: 10.1371/journal.pmed.1004737 (PMC12539716; doi:10.1371/journal.pmed.1004737)
Supplement: S6 Fig — (DOCX) [file pmed.1004737.s006.docx]

# S6 Fig. Model convergence.


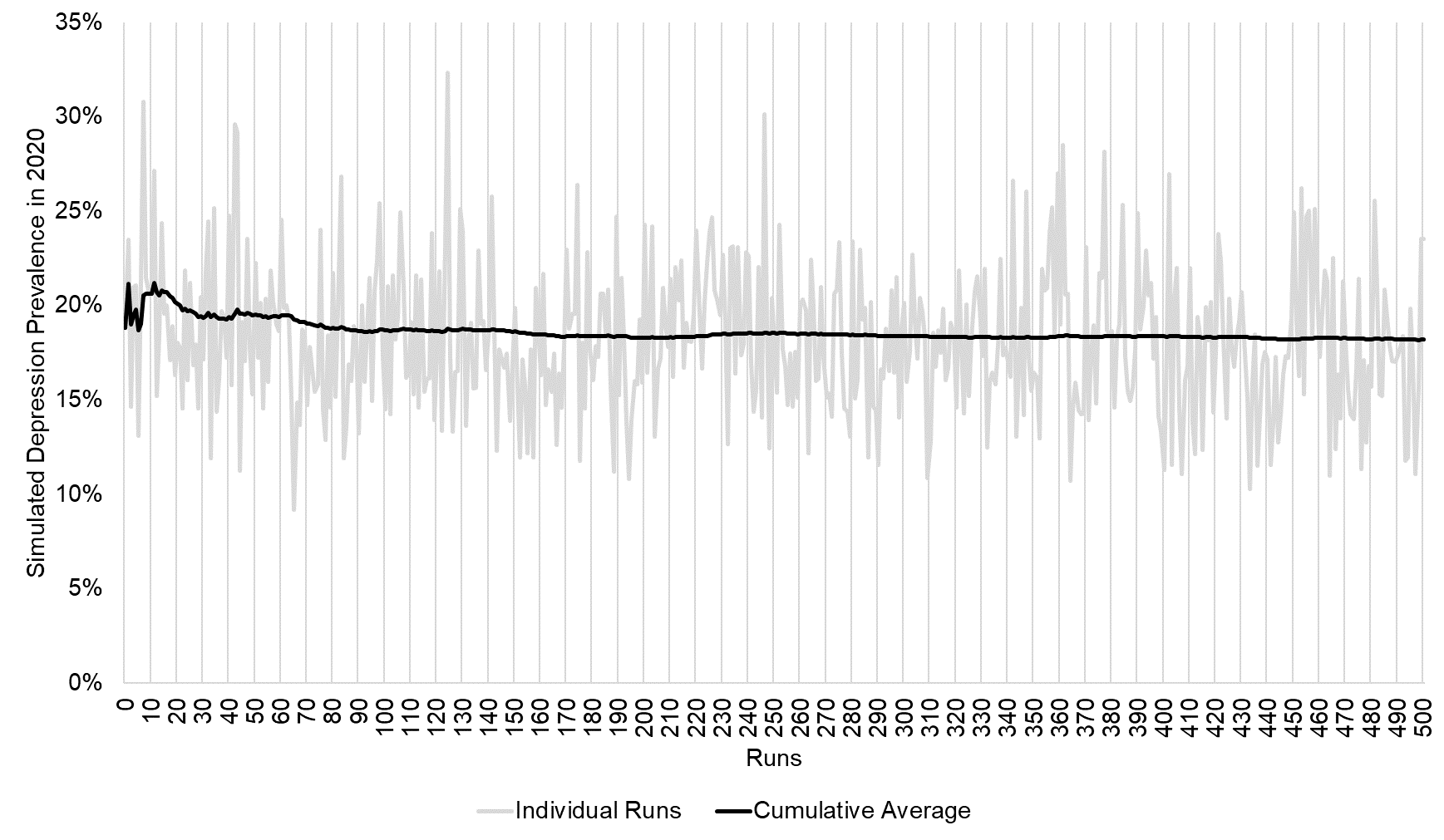


Note: The model showed good convergence, with 500 runs associated with stable estimates.
