# Supplementary material for: Impact of excessive social media use on adolescent depression and its consequences in France: An individual-based microsimulation model
Source: PLoS Med. 2025 Oct 21;22(10):e1004737. doi: 10.1371/journal.pmed.1004737 (PMC12539716; doi:10.1371/journal.pmed.1004737)
Supplement: S7 Fig — (DOCX) [file pmed.1004737.s007.docx]

# S7 Fig. Tornado diagram showing the impact of key model parameters on depression prevalence estimates.


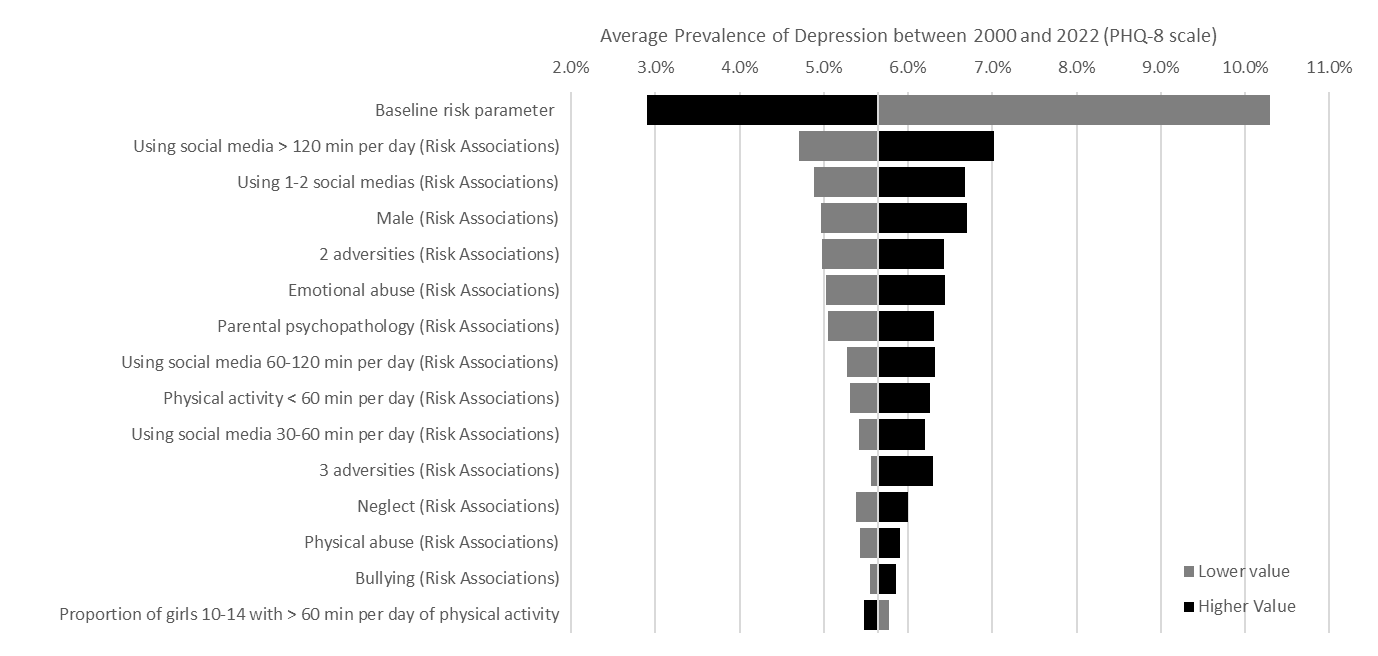


Note: The baseline risk parameter have the largest influence on outcomes, while social media and risk association parameters have moderate effects.
