# Supplementary material for: Impact of excessive social media use on adolescent depression and its consequences in France: An individual-based microsimulation model
Source: PLoS Med. 2025 Oct 21;22(10):e1004737. doi: 10.1371/journal.pmed.1004737 (PMC12539716; doi:10.1371/journal.pmed.1004737)
Supplement: S8 Fig — (DOCX) [file pmed.1004737.s008.docx]

# S8 Fig. Sensitivity analysis testing different standard deviation values (10% [A]-30% [B]) compared to baseline [C] for social media usage extrapolations.


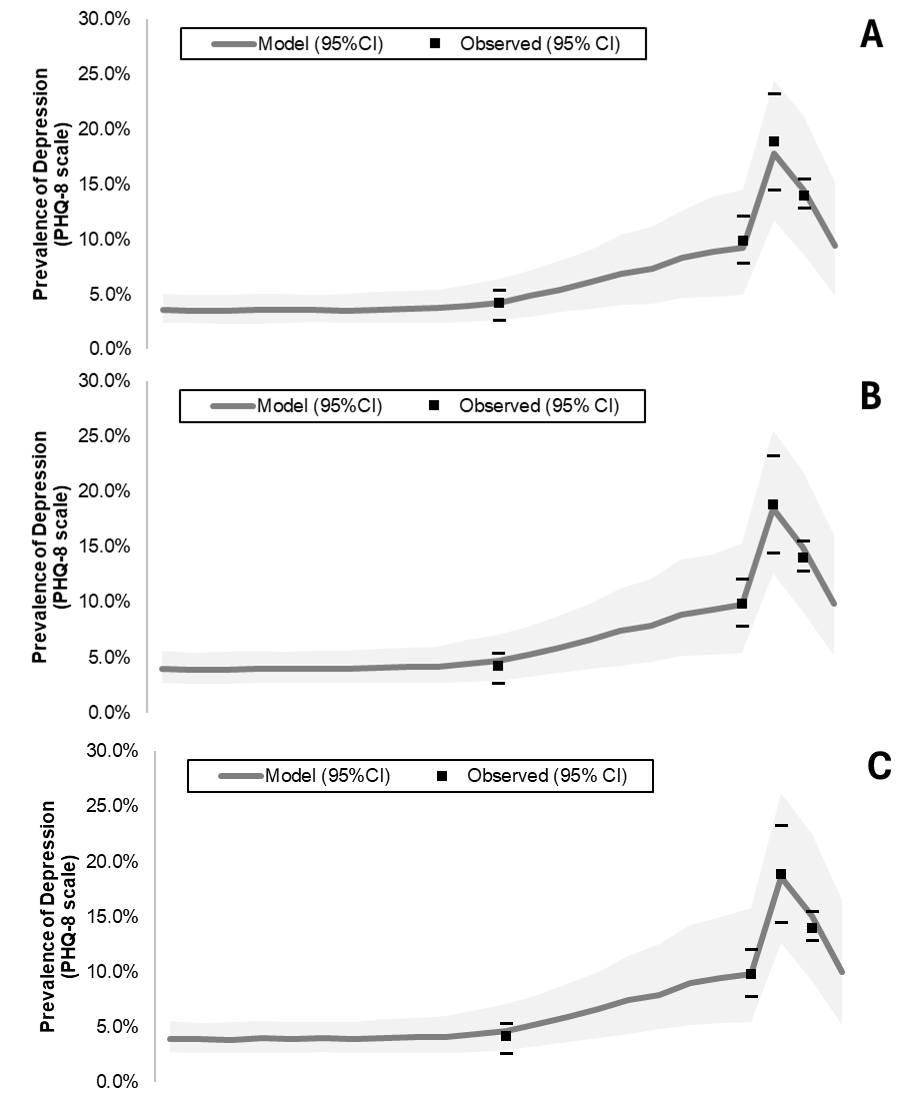


Note: Varying the standard deviation for social media usage distributions from 10% to 30% resulted in less than 5% variation in predicted depression prevalence.
