# Supplementary material for: Impact of excessive social media use on adolescent depression and its consequences in France: An individual-based microsimulation model
Source: PLoS Med. 2025 Oct 21;22(10):e1004737. doi: 10.1371/journal.pmed.1004737 (PMC12539716; doi:10.1371/journal.pmed.1004737)
Supplement: S9 Fig — (DOCX) [file pmed.1004737.s009.docx]

# S9 Fig. Sensitivity analysis testing different correlation coefficients (0% [A] -20% [B]) compared to baseline [C] between social media platforms.


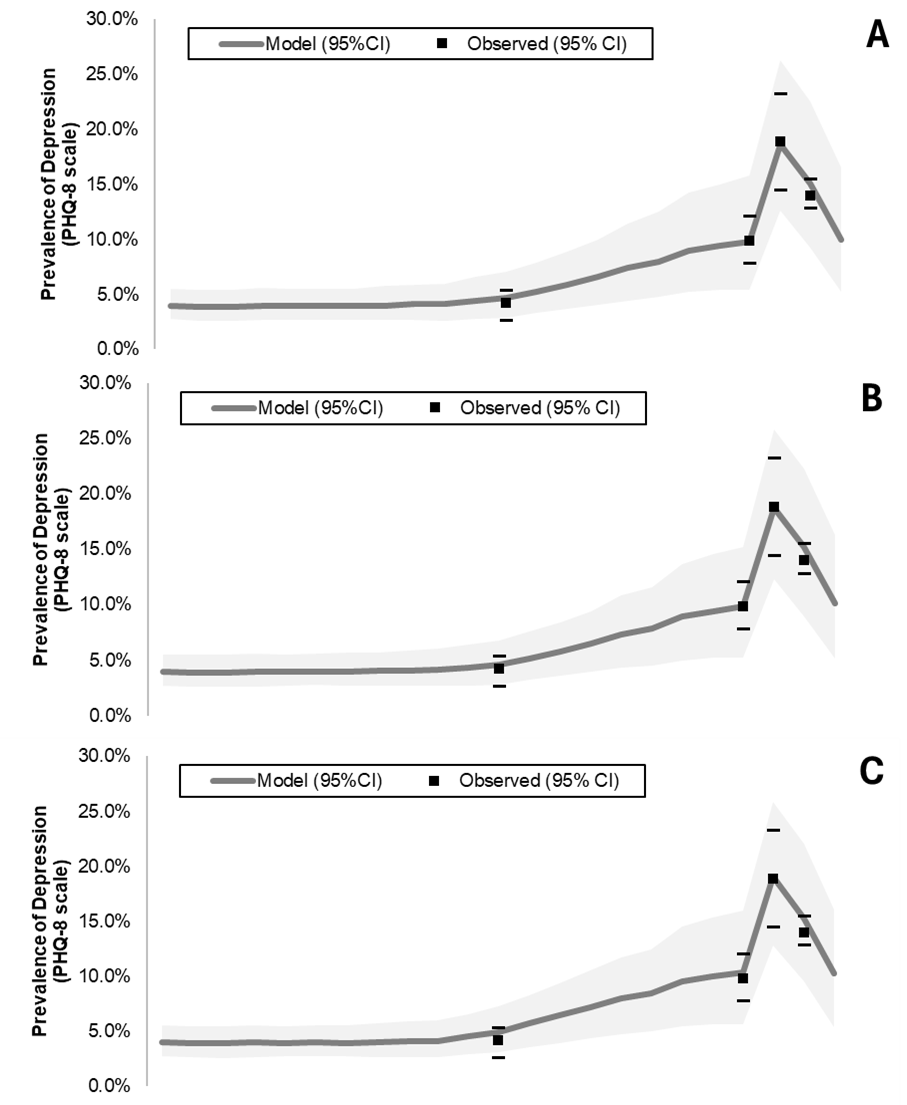


Note: Changing cross-platform correlation coefficients from 0% to 30% produced less than 5% variation in predicted depression prevalence.
