# Supplementary material for: Impact of excessive social media use on adolescent depression and its consequences in France: An individual-based microsimulation model
Source: PLoS Med. 2025 Oct 21;22(10):e1004737. doi: 10.1371/journal.pmed.1004737 (PMC12539716; doi:10.1371/journal.pmed.1004737)

# S11 Fig. Model-predicted and observed prevalence of depression across time among female and male adolescents in France if social media usage was fully restricted for the 8.5% adolescents the most at-risk for depression.

(A panel, both female and male adolescents; B panel, male adolescents; C panel, female adolescents). The solid black line represents the 'business-as-usual' calibrated trajectory showing observed trends for comparison. The shaded areas represent the 95% predicted uncertainty range stemming from the uncertainty in the parameter values.


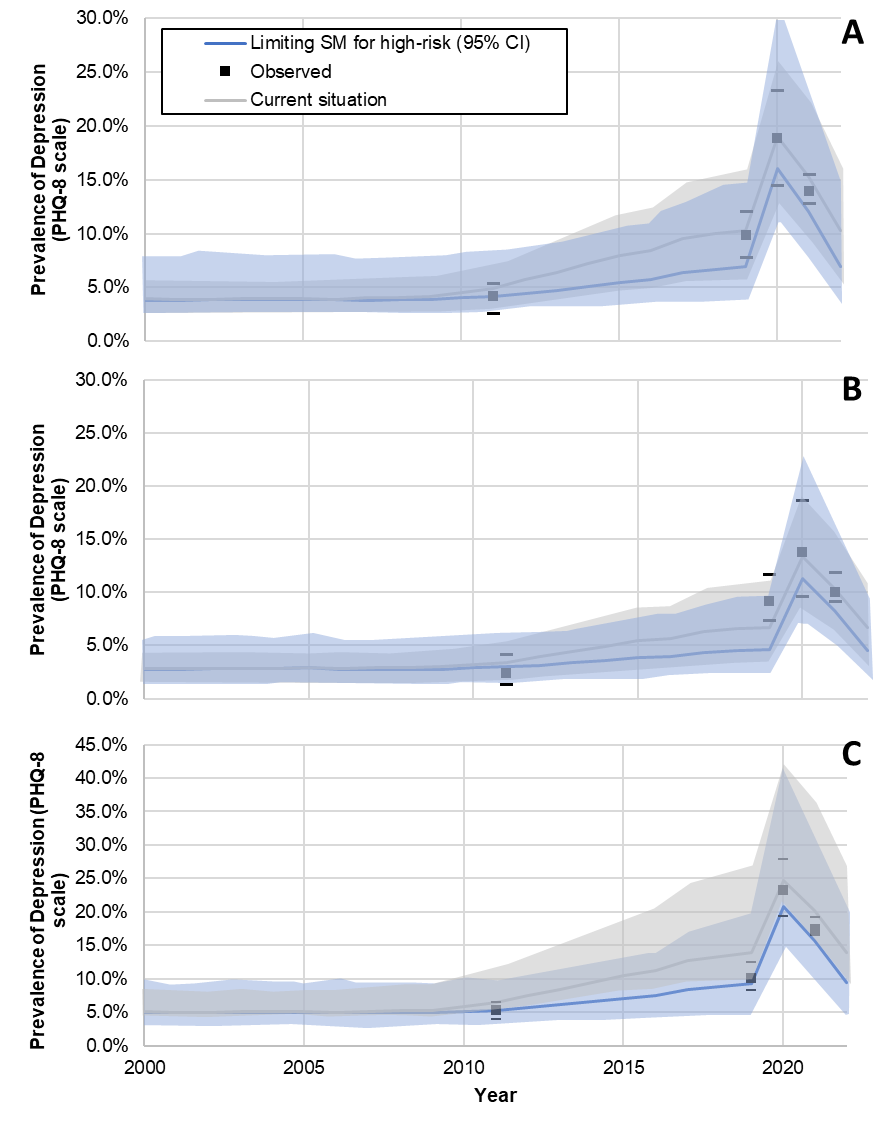

Supplement: S11 Fig — (DOCX) [file pmed.1004737.s011.docx]
