# Supplementary material for: Impact of excessive social media use on adolescent depression and its consequences in France: An individual-based microsimulation model
Source: PLoS Med. 2025 Oct 21;22(10):e1004737. doi: 10.1371/journal.pmed.1004737 (PMC12539716; doi:10.1371/journal.pmed.1004737)

# S12 Fig. Model-predicted and observed prevalence of depression across time among female and male adolescents in France if 30 min of social media usage was replaced by 30 min of physical activity from 2016 onward.

(A panel, both female and male adolescents; B panel, male adolescents; C panel, female adolescents). The solid black line represents the 'business-as-usual' calibrated trajectory showing observed trends for comparison. The shaded areas represent the 95% predicted uncertainty range stemming from the uncertainty in the parameter values.


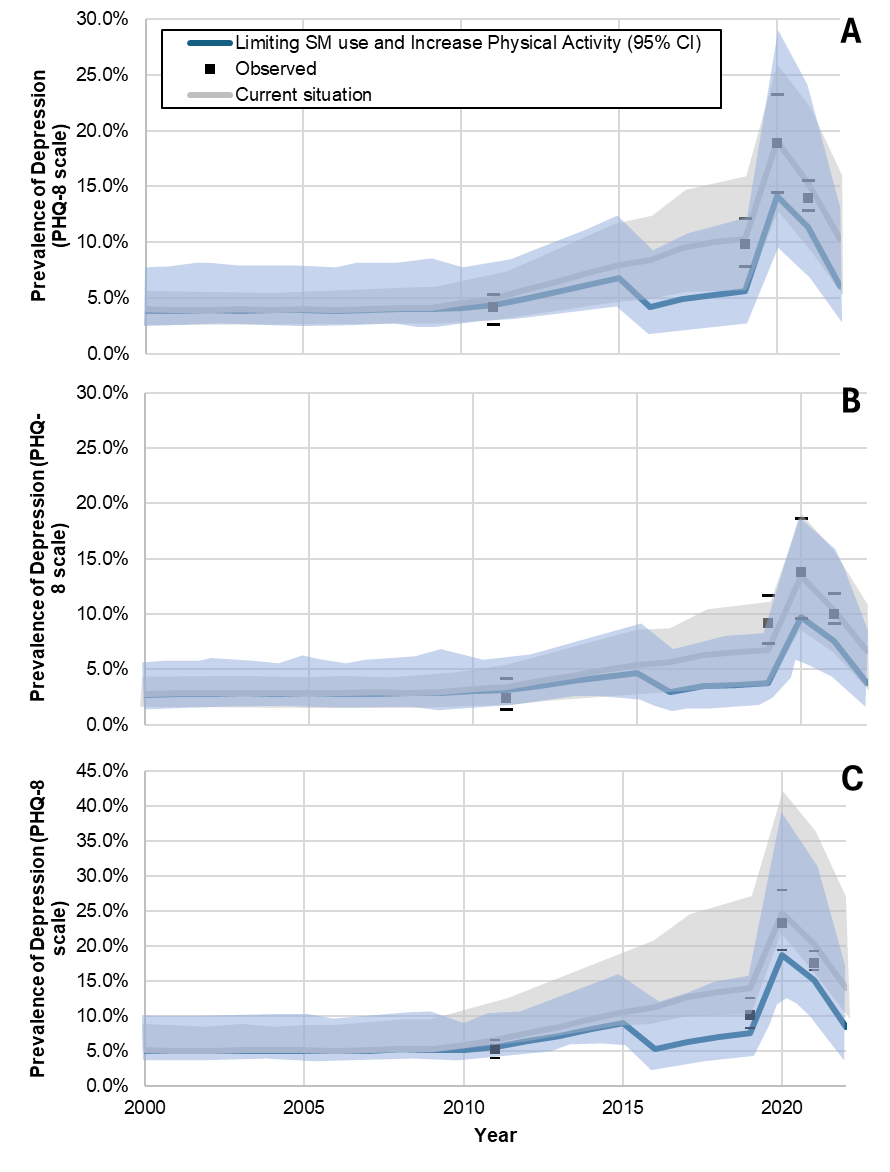

Supplement: S12 Fig — (DOCX) [file pmed.1004737.s012.docx]
