# Supplementary material for: Impact of excessive social media use on adolescent depression and its consequences in France: An individual-based microsimulation model
Source: PLoS Med. 2025 Oct 21;22(10):e1004737. doi: 10.1371/journal.pmed.1004737 (PMC12539716; doi:10.1371/journal.pmed.1004737)
Supplement: S1 Table — (DOCX) [file pmed.1004737.s013.docx]

# S1 Table. Summary of model parameters.

| **Parameters** | **Average** | **95% CI** | | **Parameter Role^a^** | **Parameter Function^b^** | **Evidence Source^c^** | **Reference number** |
| --- | --- | --- | --- | --- | --- | --- | --- |
| **Demographics and Mortality** |  |  |  |  |  |  |  |
| Background Mortality (Gompertz, alpha) | 0.1227 | 0.1227 | 0.1227 | Input | Technical | Empirical | [1] |
| Background Mortality (Gompertz, beta) | 1.993x10^-6^ | 1.990x10^-6^ | 1.993x10^-6^ | Input | Technical | Empirical | [1] |
| Background Mortality HR for calendar year (reference = 2018) | -0.0148 | -0.0148 | -0.0148 | Input | Risk association | Empirical | [1] |
| Background Mortality HR for Sex (reference = Male) | -0.0650 | -0.0650 | -0.0650 | Input | Risk association | Empirical | [1] |
| **Social Media Use Parameters (minutes)** |  |  |  |  |  |  |  |
| Average duration on Facebook | 0.1100 | 0.0900 | 0.1100 | Input | Prevalence | Empirical | [2] |
| Average duration on Twitter | 0.0400 | 0.0300 | 0.0400 | Input | Prevalence | Empirical | [2] |
| Average duration on Snapchat | 0.3400 | 0.2700 | 0.3300 | Input | Prevalence | Empirical | [2] |
| Average duration on WhatsApp | 0.0300 | 0.0200 | 0.0300 | Input | Prevalence | Empirical | [2] |
| Average duration on Instagram | 0.1100 | 0.0900 | 0.1000 | Input | Prevalence | Empirical | [2] |
| Average duration on TikTok | 0.4000 | 0.3200 | 0.3900 | Input | Prevalence | Empirical | [2] |
| Correlation between use of social medias | 0.1000 | 0.1000 | 0.1000 | Input | Technical | Expert assumption | Assumption |
| **Risk Factors Distribution** |  |  |  |  |  |  |  |
| **Childhood Adversities** |  |  |  |  |  |  |  |
| Parental psychopathology | 0.4370 | 0.3500 | 0.5240 | Input | Prevalence | Empirical | [3] |
| Physical abuse | 0.1720 | 0.1380 | 0.2060 | Input | Prevalence | Empirical | [3] |
| Emotional abuse | 0.3470 | 0.2780 | 0.4160 | Input | Prevalence | Empirical | [3] |
| Sexual abuse | 0.0440 | 0.0350 | 0.0530 | Input | Prevalence | Empirical | [3] |
| Neglect | 0.1600 | 0.1280 | 0.1920 | Input | Prevalence | Empirical | [3] |
| Bullying (Male) | 0.0790 | 0.0790 | 0.0790 | Input | Prevalence | Empirical | [4] |
| Bullying (Female) | 0.0650 | 0.0650 | 0.0650 | Input | Prevalence | Empirical | [4] |
| **Physical Activity** |  |  |  |  |  |  |  |
| Proportion of boys 10-14 with > 60 min per day of physical activity | 0.3370 | 0.2600 | 0.4240 | Input | Prevalence | Empirical | [5,6] |
| Proportion of girls 10-14 with > 60 min per day of physical activity | 0.2020 | 0.1430 | 0.2770 | Input | Prevalence | Empirical | [5,6] |
| Proportion of boys 15-17 with > 60 min per day of physical activity | 0.4010 | 0.2800 | 0.5360 | Input | Prevalence | Empirical | [5,6] |
| Proportion of girls 15-17 with > 60 min per day of physical activity | 0.1570 | 0.0970 | 0.2440 | Input | Prevalence | Empirical | [5,6] |
| Annual decrease in the number of individuals > 60 min per day of physical activity | 0.9710 | 0.8740 | 0.9616 | Input | Technical | Empirical | [7] |
| Proportion of individuals with a reduction in physical activity during COVID-19 | 0.5870 | 0.5870 | 0.5870 | Input | Prevalence | Empirical | [8] |
| **Physical Health** |  |  |  |  |  |  |  |
| **France*** |  |  |  |  |  |  |  |
| Obesity (Male) - a | 0.0184 | 0.0184 | 0.0184 | Input | Technical | Empirical | [9] |
| Obesity (Male) - b | -40.2425 | -40.2425 | -40.2425 | Input | Technical | Empirical | [9] |
| Obesity (Female) - a | 0.0289 | 0.0289 | 0.0289 | Input | Technical | Empirical | [9] |
| Obesity (Female) - b | -61.2783 | -61.2783 | -61.2783 | Input | Technical | Empirical | [9] |
| Chronic Condition (Male) - a | 0.0012 | 0.0012 | 0.0012 | Input | Technical | Empirical | [10] |
| Chronic Condition (Male) - b | -2.4139 | -2.4139 | -2.4139 | Input | Technical | Empirical | [10] |
| Chronic Condition (Female) - a | 0.0005 | 0.0005 | 0.0005 | Input | Technical | Empirical | [10] |
| Chronic Condition (Female) - b | -0.9694 | -0.9694 | -0.9694 | Input | Technical | Empirical | [10] |
| Overweight (Male) - a | -0.0015 | -0.0015 | -0.0015 | Input | Technical | Empirical | [9] |
| Overweight (Male) - b | 0.9834 | 0.9834 | 0.9834 | Input | Technical | Empirical | [9] |
| Overweight (Female) - a | 0.0099 | 0.0099 | 0.0099 | Input | Technical | Empirical | [9] |
| Overweight (Female) - b | -21.9694 | -21.9694 | -21.9694 | Input | Technical | Empirical | [9] |
| **USA** |  |  |  |  |  |  |  |
| Obesity (Male) - a | 0.0045 | 0.0045 | 0.0045 | Input | Technical | Empirical | [11] |
| Obesity (Male) - b | -8.7558 | -8.7558 | -8.7558 | Input | Technical | Empirical | [11] |
| Obesity (Female) - a | 0.0039 | 0.0039 | 0.0039 | Input | Technical | Empirical | [11] |
| Obesity (Female) - b | -7.6497 | -7.6497 | -7.6497 | Input | Technical | Empirical | [11] |
| Overweight (Male) - a | 0.0008 | 0.0008 | 0.0008 | Input | Technical | Empirical | [11] |
| Overweight (Male) - b | -1.4807 | -1.4807 | -1.4807 | Input | Technical | Empirical | [11] |
| Overweight (Female) - a | 0.0015 | 0.0015 | 0.0015 | Input | Technical | Empirical | [11] |
| Overweight (Female) - b | -2.7781 | -2.7781 | -2.7781 | Input | Technical | Empirical | [11] |
| **Substance Use** |  |  |  |  |  |  |  |
| **France*** |  |  |  |  |  |  |  |
| Alcohol (Male) - a | -0.0163 | -0.0163 | -0.0163 | Input | Technical | Empirical | [12] |
| Alcohol (Male) - b | 33.2567 | 33.2567 | 33.2567 | Input | Technical | Empirical | [12] |
| Alcohol (Female) - a | -0.0137 | -0.0137 | -0.0137 | Input | Technical | Empirical | [12] |
| Alcohol (Female) - b | 27.6360 | 27.6360 | 27.6360 | Input | Technical | Empirical | [12] |
| Tabaco (Male) - a | -0.0081 | -0.0081 | -0.0081 | Input | Technical | Empirical | [12] |
| Tabaco (Male) - b | 16.4898 | 16.4898 | 16.4898 | Input | Technical | Empirical | [12] |
| Tabaco (Female) - a | -0.0158 | -0.0158 | -0.0158 | Input | Technical | Empirical | [12] |
| Tabaco (Female) - b | 32.2271 | 32.2271 | 32.2271 | Input | Technical | Empirical | [12] |
| Cannabis (Male) - a | -0.0088 | -0.0088 | -0.0088 | Input | Technical | Empirical | [12] |
| Cannabis (Male) - b | 17.8099 | 17.8099 | 17.8099 | Input | Technical | Empirical | [12] |
| Cannabis (Female) - a | -0.0061 | -0.0061 | -0.0061 | Input | Technical | Empirical | [12] |
| Cannabis (Female) - b | 12.3673 | 12.3673 | 12.3673 | Input | Technical | Empirical | [12] |
| **USA*** |  |  |  |  |  |  |  |
| Alcohol (Male) - a | -0.0115 | -0.0115 | -0.0115 | Input | Technical | Empirical | [13] |
| Alcohol (Male) - b | 23.4515 | 23.4515 | 23.4515 | Input | Technical | Empirical | [13] |
| Alcohol (Female) - a | -0.0115 | -0.0115 | -0.0115 | Input | Technical | Empirical | [13] |
| Alcohol (Female) - b | 23.4515 | 23.4515 | 23.4515 | Input | Technical | Empirical | [13] |
| Tabaco (Male) - a | -0.0057 | -0.0057 | -0.0057 | Input | Technical | Empirical | [13] |
| Tabaco (Male) - b | 11.5950 | 11.5950 | 11.5950 | Input | Technical | Empirical | [13] |
| Tabaco (Female) - a | -0.0057 | -0.0057 | -0.0057 | Input | Technical | Empirical | [13] |
| Tabaco (Female) - b | 11.5950 | 11.5950 | 11.5950 | Input | Technical | Empirical | [13] |
| Cannabis (Male) - a | -0.0034 | -0.0034 | -0.0034 | Input | Technical | Empirical | [13] |
| Cannabis (Male) - b | 7.2005 | 7.2005 | 7.2005 | Input | Technical | Empirical | [13] |
| Cannabis (Female) - a | -0.0034 | -0.0034 | -0.0034 | Input | Technical | Empirical | [13] |
| Cannabis (Female) - b | 7.2005 | 7.2005 | 7.2005 | Input | Technical | Empirical | [13] |
| **Depression Risk Associations [ln(OR)]** |  |  |  |  |  |  |  |
| **Calibrated Parameter** |  |  |  |  |  |  |  |
| Baseline risk of depression | 1.8000 | 1.8000 | 1.8000 | Calibrated | Calibration | Model fitting | - |
| **Childhood Adversities Associations** |  |  |  |  |  |  |  |
| Parental psychopathology | -0.0862 | -0.2151 | 0.0513 | Input | Risk association | Empirical | [14] |
| Physical abuse | 0.1278 | -0.0100 | 0.2614 | Input | Risk association | Empirical | [14] |
| Emotional abuse | -0.2852 | -0.4318 | -0.1398 | Input | Risk association | Empirical | [14] |
| Sexual abuse | -0.0392 | -0.2311 | 0.1508 | Input | Risk association | Empirical | [14] |
| Neglect | -0.0862 | -0.2231 | 0.0408 | Input | Risk association | Empirical | [14] |
| Bullying | -0.4383 | -0.5710 | -0.3001 | Input | Risk association | Empirical | [14] |
| 2 adversities | 0.0000 | -0.1655 | 0.1744 | Input | Risk association | Empirical | [14] |
| 3 adversities | 0.0000 | -0.2546 | 0.0741 | Input | Risk association | Empirical | [14] |
| 4+ adversities | 0.3425 | -0.0100 | 0.4649 | Input | Risk association | Empirical | [14] |
| **Social Media Use Associations** |  |  |  |  |  |  |  |
| Using 1-2 social medias | -0.4511 | -0.8154 | -0.0770 | Input | Risk association | Empirical | [15] |
| Using 3-4 social medias | -0.7701 | -1.2030 | -0.3365 | Input | Risk association | Empirical | [15] |
| Using > 4 social medias | -1.1249 | -1.6074 | -0.6471 | Input | Risk association | Empirical | [15] |
| Using social media 30-60 min per day | 0.0513 | -0.3507 | 0.4620 | Input | Risk association | Empirical | [15] |
| Using social media 60-120 min per day | -0.2776 | -0.6881 | 0.1278 | Input | Risk association | Empirical | [15] |
| Using social media > 120 min per day | -0.1823 | -0.5766 | 0.2107 | Input | Risk association | Empirical | [15] |
| **Lifestyle and Health Associations** |  |  |  |  |  |  |  |
| Physical activity < 60 min per day | 0.4780 | 0.1278 | 0.8210 | Input | Risk association | Empirical | [16] |
| Overweight | 0.0392 | -0.0513 | 0.1310 | Input | Risk association | Empirical | [17,18] |
| Obesity (Male) | 0.0770 | -0.1625 | 0.3148 | Input | Risk association | Empirical | [17,18] |
| Obesity (Female) | 0.3646 | 0.1823 | 0.5423 | Input | Risk association | Empirical | [17,18] |
| Chronic Condition | 0.3852 | 0.1620 | 0.5675 | Input | Risk association | Empirical | [19] |
| **Substance Use Associations** |  |  |  |  |  |  |  |
| Cannabis Use | 0.2852 | 0.1740 | 0.3988 | Input | Risk association | Empirical | [20] |
| Tabaco Use | 0.5933 | 0.2070 | 1.0473 | Input | Risk association | Empirical | [20] |
| Alcohol Use | 0.4318 | 0.1740 | 0.6931 | Input | Risk association | Empirical | [20] |
| **Demographic and Contextual Associations** |  |  |  |  |  |  |  |
| Male | 0.2357 | -0.0392 | 0.4943 | Input | Risk association | Empirical | [15] |
| COVID19 | -1.4816 | -1.6864 | -1.2809 | Input | Risk association | Empirical | [21] |

**^a^** Input (external sources) vs. Calibrated (model-fitted).

**^b^** Prevalence (population rates), Risk association (odds/hazard ratios), Technical (mathematical coefficients), Calibration (fitted parameter).

**^c^** Empirical (primary studies), Expert assumption (professional judgment), Model fitting (calibration-derived).

# References

1. INSEE. Décès et taux de mortalité - Données annuelles de 1982 à 2023. 2024. doi:https://www.insee.fr/fr/statistiques/2383440

2. Asselin C. Les réseaux sociaux en France et dans le monde : les chiffres d’utilisation en 2021. In: DIGIMIND. Médiamétrie et Médiamétrie/NetRatings. [Internet]. 21 Apr 2021 [cited 25 Aug 2023]. Available: https://blog.digimind.com/fr/tendances/r%C3%A9seaux-sociaux-france-monde-chiffres-utilisation-2021

3. Biscond M, Revranche M, Navarro-Mateu F, Janota M, Kovess-Masfety V, Husky MM. The effect of childhood adversities on the persistence of suicidal ideation and plans among college students: A longitudinal study. Journal of Affective Disorders. 2023;323: 354–360. doi:10.1016/j.jad.2022.11.078

4. Léon C, Spilka S, Ehlinger V, Godeau E. Santé mentale et bien-être : Résultats de l’Enquête nationale en collèges et en lycées chez les adolescents sur la santé et les substances - EnCLASS 2018. Santé Publique France; 2021 Jun. Available: https://www.santepubliquefrance.fr/maladies-et-traumatismes/sante-mentale/depression-et-anxiete/documents/rapport-synthese/sante-mentale-et-bien-etre-resultats-de-l-enquete-nationale-en-colleges-et-en-lycees-chez-les-adolescents-sur-la-sante-et-les-substances-enclas

5. Verdot C, Salanave B, Deschamps V. Activité physique et sédentarité dans la population française. Situation en 2014-2016 et évolution depuis 2006-2007. Paris: Santé publique France; 2020 Jun pp. 296–304. Report No.: N^o^ 15. Available: http://beh.santepublique france.fr/beh/2020/15/2020_15_1.html

6. Van Sluijs EMF, Ekelund U, Crochemore-Silva I, Guthold R, Ha A, Lubans D, et al. Physical activity behaviours in adolescence: current evidence and opportunities for intervention. The Lancet. 2021;398: 429–442. doi:10.1016/S0140-6736(21)01259-9

7. Inchley J, Currie D, Budisavljevic S, Torsheim T, Jåstad A, Cosma A, et al. Spotlight on adolescent health and well-being. Findings from the 2017/2018 Health Behaviour in School-aged Children (HBSC) survey in Europe and Canada. International report. Volume 1. Key findings. Copenhagen: WHO Regional Office for Europe; 2020. Report No.: ISBN 978 92 890 5500 0. Available: https://www.who.int/europe/publications/i/item/9789289055000

8. Chambonniere C, Lambert C, Fearnbach N, Tardieu M, Fillon A, Genin P, et al. Effect of the COVID-19 lockdown on physical activity and sedentary behaviors in French children and adolescents: New results from the ONAPS national survey. European Journal of Integrative Medicine. 2021;43: 101308. doi:10.1016/j.eujim.2021.101308

9. Guignon N. En 2017, des adolescents plutôt en meilleure santé physique mais plus souvent en surcharge pondérale. DREES; 2019 Aug. Report No.: N° 1122. Available: https://drees.solidarites-sante.gouv.fr/publications/etudes-et-resultats/en-2017-des-adolescents-plutot-en-meilleure-sante-physique-mais

10. Coste J, Mandereau-Bruno L, Carcaillon-Bentata L. Identification des maladies chroniques dans le Système national des données de santé : recensement des algorithmes publiés et faisabilité de leur implémentation pour la surveillance épidémiologique. Revue d’Épidémiologie et de Santé Publique. 2022;70: S27. doi:10.1016/j.respe.2022.01.110

11. Fryar CD, Carroll MD, Afful J. Prevalence of Overweight, Obesity, and Severe Obesity Among Children and Adolescents Aged 2–19 Years: United States, 1963–1965 Through 2017–2018. USA: CDC National Center for Health Statistics; 2021 Jan. Available: https://www.cdc.gov/nchs/data/hestat/obesity-child-17-18/obesity-child.htm#Suggested%20citation

12. Spilka S, Godeau E, Nézet O, Ehlinger V, Janssen E. Usages d’alcool, de tabac et de cannabis chez les adolescents du secondaire en 2018. OFDT; 2019 Jun. Report No.: N^o^ 132.

13. Ball J, Grucza R, Livingston M, Ter Bogt T, Currie C, De Looze M. The great decline in adolescent risk behaviours: Unitary trend, separate trends, or cascade? Social Science & Medicine. 2023;317: 115616. doi:10.1016/j.socscimed.2022.115616

14. Husky MM, Sadikova E, Lee S, Alonso J, Auerbach RP, Bantjes J, et al. Childhood adversities and mental disorders in first-year college students: results from the World Mental Health International College Student Initiative. Psychol Med. 2023;53: 2963–2973. doi:10.1017/S0033291721004980

15. Primack BA, Shensa A, Sidani JE, Whaite EO, Lin LY, Rosen D, et al. Social Media Use and Perceived Social Isolation Among Young Adults in the U.S. American Journal of Preventive Medicine. 2017;53: 1–8. doi:10.1016/j.amepre.2017.01.010

16. Kremer P, Elshaug C, Leslie E, Toumbourou JW, Patton GC, Williams J. Physical activity, leisure-time screen use and depression among children and young adolescents. Journal of Science and Medicine in Sport. 2014;17: 183–187. doi:10.1016/j.jsams.2013.03.012

17. Sutaria S, Devakumar D, Yasuda SS, Das S, Saxena S. Is obesity associated with depression in children? Systematic review and meta-analysis. Arch Dis Child. 2019;104: 64–74. doi:10.1136/archdischild-2017-314608

18. Luppino FS, De Wit LM, Bouvy PF, Stijnen T, Cuijpers P, Penninx BWJH, et al. Overweight, Obesity, and Depression: A Systematic Review and Meta-analysis of Longitudinal Studies. Arch Gen Psychiatry. 2010;67: 220. doi:10.1001/archgenpsychiatry.2010.2

19. Pinquart M, Shen Y. Depressive Symptoms in Children and Adolescents with Chronic Physical Illness: An Updated Meta-Analysis. Journal of Pediatric Psychology. 2011;36: 375–384. doi:10.1093/jpepsy/jsq104

20. Esmaeelzadeh S, Moraros J, Thorpe L, Bird Y. Examining the Association and Directionality between Mental Health Disorders and Substance Use among Adolescents and Young Adults in the U.S. and Canada—A Systematic Review and Meta-Analysis. JCM. 2018;7: 543. doi:10.3390/jcm7120543

21. Léon C. Prévalence des épisodes dépressifs en France chez les 18-85 ans : résultats du Baromètre santé 2021. Saint-Maurice: Santé publique France; 2023 Feb pp. 28–40. Report No.: N^o^ 2. Available: https://www.santepubliquefrance.fr/maladies-et-traumatismes/sante-mentale/depression-et-anxiete/documents/article/prevalence-des-episodes-depressifs-en-france-chez-les-18-85-ans-resultats-du-barometre-sante-2021
