# Supplementary material for: Impact of excessive social media use on adolescent depression and its consequences in France: An individual-based microsimulation model
Source: PLoS Med. 2025 Oct 21;22(10):e1004737. doi: 10.1371/journal.pmed.1004737 (PMC12539716; doi:10.1371/journal.pmed.1004737)
Supplement: S2 Table — (DOCX) [file pmed.1004737.s014.docx]

# S2 Table. Projected average time spent by adolescents on Social Media, by media.

| **Social Media** | **Average Duration per Day (hours)** | **Introduction Year** | **Peak Usage Period** |
| --- | --- | --- | --- |
| Facebook | 0.69 | 2004 | 2017-2021 |
| Twitter | 0.24 | 2006 | 2017-2021 |
| Snapchat | 2.14 | 2011 | 2019-2023 |
| WhatsApp | 0.19 | 2009 | 2020-2021 |
| Instagram | 0.68 | 2010 | 2016-2020 |
| TikTok | 2.52 | 2016 | 2018-2021 |

Note: Peak usage periods were derived from modeled adoption curves (S1 and S2 Figures). Longitudinal trends were captured using gamma distribution extrapolation to model temporal variations influencing depression risk across birth cohorts.
