# Supplementary material for: Impact of excessive social media use on adolescent depression and its consequences in France: An individual-based microsimulation model
Source: PLoS Med. 2025 Oct 21;22(10):e1004737. doi: 10.1371/journal.pmed.1004737 (PMC12539716; doi:10.1371/journal.pmed.1004737)
Supplement: Attachment — Submitted filename: response to reviewers.docx [file pmed.1004737.s018.docx]

July 8th, 2025,

Dear Editor,

Thank you very much for giving us the opportunity to submit a revised version of our manuscript PMEDICINE-D-24-03990R1 “An Individual-Based Microsimulation Model of the Impact of Excessive Social Media Use On Adolescent Depression and Its Consequences in France” to be considered for publication in PLOS Medicine.

We appreciate the time and effort that you and the reviewers have dedicated in providing valuable feedback on our manuscript. A point-by-point response is detailed below, and we have highlighted the corresponding changes in yellow within the manuscript.

The manuscript contains no data, patient information, or other material or results that have been published elsewhere. It has not been submitted to any other journal, nor have the results been presented at any scientific meeting.

We thank you in advance for considering this contribution.

Nicolas HOERTEL, MD, MPH, Ph.D.,

On behalf of all co-authors,

Université Paris Cité, AP-HP Centre, DMU Psychiatrie et Addictologie,

Corentin Celton Hospital, Issy-les-Moulineaux, France

# Reviewer #1

## 1/ Major Comments. The microsimulation model structure lacks transparency. Providing detailed justifications for key assumptions, particularly the calibration of residual baseline risk, would enhance the credibility of the model.

Answer: We thank the reviewer for giving us the opportunity to clarify the microsimulation model structure. We have expanded the methods section to provide detailed justification for the calibration approach, particularly explaining that only one parameter (baseline risk) required calibration, one parameter (correlation between use of social medias) was assumed, while all others were literature-based. We modified the manuscript as follows (please see also Supplementary Table 1):

*P5-6: “Following previously described methods [29–33], we built an individual-based microsimulation model to test the hypothesis that social media (SM) use increases the risk of depression among adolescents. This model comprised 95 parameters grouped into three modules: (1) demographic characteristics and mortality (4 parameters), (2) social media use (7 parameters), and (3) risk factors for adolescent depression (84 parameters). Of these 95 parameters, 93 were derived directly from published literature, one was based on expert judgment, while only one parameter—the individual baseline risk—required calibration. Detailed values for all parameters and the references used to estimate their values can be found in S1 Table.”*

*P10-11: “2.6. Model calibration*

*Of the 95 model parameters, 93 were obtained from prior studies, one was based on expert judgment, while one parameter, the individual baseline risk of depression, could not be estimated from the literature and was therefore calibrated (S1 Table). This baseline risk represents all unmeasured factors contributing to depression risk beyond the documented risk factors in our model. The calibration targeted the observed depression prevalence in France from l'Enquête santé européenne (EHIS) [74] and l'Enquête nationale Épidémiologie et Conditions de vie (EpiCov) (Inserm-DREES) [75] that included 85,000 people aged 15 years or more who responded to the Patient Health Questionnaire depression scale (PHQ-9) [76]. Individuals with a projected probability of depression higher than 50% were considered as having depression in the simulation model (S3 Figure). We used the Nelder-Mead simplex method [77] with convergence after 300 iterations or when the loss function fell below 0.001. The calibration process was repeated 30 times with different initial values to avoid local minima. Consistent results were observed across all 30 calibration runs, with the loss function consistently converging, suggesting robustness of the calibration process. The final calibrated baseline risk (-1.80 (SD=1.0)) was applied to both sexes.”*

*P11: “The microsimulation model was run on 1,000 individuals per birth cohort from 1990 to 2012. This sample size provides adequate statistical power for the primary outcome (depression prevalence) while maintaining computational feasibility. Although this may introduce sampling variability for rare outcomes such as suicide, our Monte Carlo approach with 500 iterations based on the random variation of all non-calibrated parameters simultaneously, either within a 95% confidence interval (CI) for parameters estimated from the literature or within a ±20% interval when CIs were not available, allows us to capture this uncertainty in the confidence intervals presented. This Monte Carlo approach propagated parameter uncertainty by independently sampling all 94 non-calibrated parameters in each iteration and running the model forward to observe output variability, capturing uncertainty across all model predictions.”*

## 2/ The model assumes additive effects of risk factors, potentially ignoring synergistic or multiplicative interactions (e.g., how childhood adversity combined with excessive social media use could amplify depression risk). Including interaction terms or testing these assumptions would improve realism. The risk equation assumes a log-linear relationship between risk factors and depression, potentially oversimplifying interactions. Testing for non-linearities or complex interactions among key risk factors could improve accuracy.

Answer: We acknowledge this important limitation. Our additive log-linear model was chosen for parsimony and stability, as insufficient empirical evidence exists to reliably parameterize specific interactions. Following this comment, we have expanded the limitations section as follows:

*P18: "**Second, our model assumes additive effects of risk factors, and does not include interaction terms between risk factors, preventing it from capturing potential synergistic effects (e.g., between childhood adversity and social media use) or suppression effects. This additive log-linear model was chosen for parsimony and stability, as insufficient empirical evidence exists to reliably parameterize specific interactions. However, this approach is likely to have oversimplified non-linear associations between individual risk factors and depression."*

## 3/ Extrapolating social media usage with a gamma distribution assumes consistent patterns across platforms and demographic groups, which may not hold. Validation of these assumptions against independent datasets is recommended.

Answer: We thank the reviewer for this comment. We agree that platform and demographic heterogeneity exists. The gamma distribution was chosen based on its established use in modeling positively skewed usage data. Our validation against both French and US datasets support this assumption. We have clarified this choice and added appropriate citations. Following this comment, we have revised the manuscript as follows:

*P6: “Daily usage durations per platform were estimated based on specific data from France [37] and were extrapolated using a gamma distribution, which appropriately captures the right-skewed nature of social media usage where most users have moderate usage while some have very high usage [39], assuming a coefficient of variation (SD/mean) of 20%. The gamma distribution parameters were applied uniformly across platforms as a simplifying assumption, even if platform-specific distributions may exist.”*

## 4/ Validation against U.S. data adds robustness, but the assumption that other risk factors are similarly distributed between France and the U.S. needs stronger justification. Regional variations in mental health care access and cultural attitudes may limit generalisability.

Answer: We agree that cross-country differences may exist. However, in our model validation against US data, we recalibrated the baseline depression risk, capturing potential regional variations related to care access or cultural attitudes, and incorporated US-specific values for obesity and substance use. We assumed comparable distributions for remaining risk factors. However, we now acknowledge this limitation regarding cross-country assumptions and have modified the manuscript accordingly:

*P19-20: “Finally, while our US validation model recalibrated baseline depression risk and used US-specific data for obesity and substance use, we assumed comparable distributions for other risk factors between countries. Regional variations in mental health care access, cultural attitudes toward mental health, family structures, and social media usage patterns may limit the generalizability of these assumptions. However, the adequate validation against US data suggests that the core modeled relationships may be sufficiently robust to capture depression trends across different healthcare and social systems, at least for high-income countries with similar social media penetration.”*

## 5/ The calibration process (Nelder-Mead simplex method) is standard but susceptible to local minima. Conducting sensitivity analyses to explore how variations in calibration parameters influence outcomes would strengthen the findings.

Answer: We agree with the reviewer. We have performed 30 runs of the calibration process to reduce the risk of local minima and have observed consistent convergence. We have clarified this point in the methods and have added that the loss function consistently converged, suggesting robustness.

*P10-11: “Consistent results were observed across all 30 calibration runs, with the loss function consistently converging, suggesting robustness of the calibration process.”*

**6/ For bootstrapped samples, it is unclear whether convergence diagnostics were performed to ensure stability of estimates. This should be explicitly addressed. Bootstrapping may underestimate uncertainty when parameter distributions are highly skewed or non-normal. Alternative methods, such as Monte Carlo simulations, should be considered to verify robustness. The reliance on bootstrapping lacks evaluation of potential biases from non-independence in samples. Addressing this limitation or testing alternative resampling methods would strengthen the findings.**

Answer: We thank the reviewer for this important observation. We acknowledge that the term "bootstrapping" was used inappropriately in the original manuscript. The simulations relied on a Monte Carlo approach in which all non-calibrated parameters were randomly drawn from predefined distributions across 500 iterations. No convergence diagnostics were performed, as the baseline risk remained fixed across simulations, and no iterative optimization was involved in this stage. The Monte Carlo approach with independent parameter sampling from predefined distributions avoids the non-independence issues associated with traditional bootstrapping methods highlighted by the reviewer.

*P11: “The microsimulation model was run on 1,000 individuals per birth cohort from 1990 to 2012. This sample size provides adequate statistical power for the primary outcome (depression prevalence) while maintaining computational feasibility. Although this may introduce sampling variability for rare outcomes such as suicide, our Monte Carlo approach with 500 iterations based on the random variation of all non-calibrated parameters simultaneously, either within a 95% confidence interval (CI) for parameters estimated from the literature or within a ±20% interval when CIs were not available, allows us to capture this uncertainty in the confidence intervals presented. This Monte Carlo approach propagated parameter uncertainty by independently sampling all 94 non-calibrated parameters in each iteration and running the model forward to observe output variability, capturing uncertainty across all model predictions. Parameters were independently sampled from predefined distributions in each simulation, distinguishing this from bootstrapping. Parameter distributions were selected based on the nature of the variable: normal distributions for continuous variables with central tendency (e.g., baseline risk), uniform for bounded parameters without clear central values, and empirical distributions when preserved from literature. No convergence diagnostics were needed as baseline risk remained fixed. Average results and uncertainty were based on the average and distribution of the 500 Monte Carlo simulations. Results were extrapolated to the French general population exposed to SM, i.e. aged 10 to 17 years between 1990 et 2012, based on an average of 790,000 birth per year between 1990 and 2012.”*

## 7/ Beyond R² and Pearson's R, metrics such as mean absolute error (MAE) or root mean square error (RMSE) could provide a more nuanced assessment of model fit.

Answer: Following this comment, we have added additional model fit metrics, including MAE and RMSE, to provide a more comprehensive assessment. We modified the manuscript as follows:

*P12: “Additionally, residual plots were generated to evaluate systematic biases and temporal patterns in prediction errors, and diagnostic plots were constructed to assess key model assumptions including independence of residuals over time.”*

*P13: “Data were collected using Microsoft Excel. The model was performed using C++ and statistical analyses were conducted using SAS software version 9.4. The threshold for statistical significance was a priori fixed at two-sided p<0.05. However, we prioritized effect sizes and confidence intervals over p-values, and presented R² and Pearson's R, as well as Mean Absolute Error (MAE) and Root Mean Square Error (RMSE) following current statistical practices [82,83].”*

*P14: “Residual analysis showed no systematic bias patterns, and diagnostic plots confirmed adequate model assumptions (temporal independence) (S5 Figure). Mean Absolute Error (MAE) and Root Mean Square Error (RMSE) were, respectively, 0.21 and 0.27 for SM use, and 0.005 and 0.006 for depression rates. ”*

## 8/ The model simulates annual risk assessments but does not account for intra-annual variations (e.g., seasonal effects on social media use or depression rates). Exploring these could offer more granular insights.

Answer: We now acknowledge that seasonality could influence depression and social media use, but we lacked reliable monthly data to model this robustly. We opted for annual risk estimates to maintain model parsimony and avoid overfitting. Following this comment, we have added this point to the discussion as follows:

*P18: “Fourth, our model did not account for seasonal variations in social media use or depression rates, potentially missing intra-annual patterns, in the absence of reliable monthly data.”*

## 9/ Some parameters (e.g., the 20% standard deviation for social media usage extrapolations) appear arbitrary. Providing sensitivity analyses or citing empirical evidence for these choices would improve confidence in the model. The paper mentions uncertainty intervals but lacks detail on how parameter uncertainty propagates through the model. Bayesian approaches or probabilistic sensitivity analyses might better capture full uncertainty.

Answer: We appreciate the reviewer's valuable comments regarding parameter uncertainty and sensitivity analyses. Regarding the concern about the 20% standard deviation for social media usage extrapolations: this coefficient of variation was selected as a conservative estimate based on typical patterns observed in social media usage data (<https://www.mediametrie.fr/>). To test the robustness of this assumption, we have conducted systematic sensitivity analyses examining the impact of varying this parameter. We have also clarified our approach to uncertainty propagation. What was previously described as "bootstrapping" was in fact a Monte Carlo simulation approach. We have revised our description on Page 10. We have further elaborated on our parameter distribution selection. Finally, while we acknowledge that Bayesian approaches might offer alternative methods for uncertainty quantification, our Monte Carlo simulation approach is well-established in microsimulation modeling and effectively captures parameter uncertainty while maintaining computational feasibility. The sensitivity analyses demonstrate that our results are robust across a range of parameter values, providing confidence in the model's findings despite the inherent uncertainty in some parameter selections. The supplementary materials now include tornado diagrams and sensitivity analysis results, providing transparent documentation of how parameter variations affect model outcomes.

This assumption was selected based on empirical coefficient of variation in modeling approach and intended as a conservative estimate. We now clarify this in the methods and include it in sensitivity analyses.

*P11: “The microsimulation model was run on 1,000 individuals per birth cohort from 1990 to 2012. This sample size provides adequate statistical power for the primary outcome (depression prevalence) while maintaining computational feasibility. Although this may introduce sampling variability for rare outcomes such as suicide, our Monte Carlo approach with 500 iterations based on the random variation of all non-calibrated parameters simultaneously, either within a 95% confidence interval (CI) for parameters estimated from the literature or within a ±20% interval when CIs were not available, allows us to capture this uncertainty in the confidence intervals presented. This Monte Carlo approach propagated parameter uncertainty by independently sampling all 94 non-calibrated parameters in each iteration and running the model forward to observe output variability, capturing uncertainty across all model predictions. Parameters were independently sampled from predefined distributions in each simulation, distinguishing this from bootstrapping. Parameter distributions were selected based on the nature of the variable: normal distributions for continuous variables with central tendency (e.g., baseline risk), uniform for bounded parameters without clear central values, and empirical distributions when preserved from literature. No convergence diagnostics were needed as baseline risk remained fixed. Average results and uncertainty were based on the average and distribution of the 500 Monte Carlo simulations. Results were extrapolated to the French general population exposed to SM, i.e. aged 10 to 17 years between 1990 et 2012, based on an average of 790,000 birth per year between 1990 and 2012.”*

*P12: “Additionally, residual plots were generated to evaluate systematic biases and temporal patterns in prediction errors, and diagnostic plots were constructed to assess key model assumptions including independence of residuals over time. We also performed three sensitivity analyses to assess model robustness: (1) tornado diagrams examining the individual impact of each key parameter on depression prevalence estimates, (2) testing alternative standard deviation values (10% and 30%, instead of 20%) for the gamma distribution used in social media usage extrapolations, and (3) testing different correlation coefficients (0.00 and 0.20, instead of 0.10) for cross-platform social media use. Results from all sensitivity analyses are reported with their impact on the primary outcome.”*

*P14: “Sensitivity analyses demonstrated model robustness across all tested parameters. Tornado diagrams (S7 Figure) showed that the baseline risk parameter had the largest influence on outcomes, while social media and risk factor parameters had moderate effects. Varying the standard deviation for social media usage distributions from 10% to 30% resulted in less than 5% variation in predicted depression prevalence (S8 Figure). Similarly, changing cross-platform correlation coefficients from 0% to 20% produced less than 5% variation in predicted depression prevalence (S9 Figure).”*

## 10/ For statistical significance reporting effect sizes, confidence intervals, and advanced model fit metrics instead of p<0.05 would align better with current statistical practices.

Answer: We fully agree that effect sizes, confidence intervals, and comprehensive model fit metrics provide more informative assessments than p-values alone. Following this comment, we have enhanced our statistical reporting approach to align with current best practices, as follows (please see also responses to comments #7 and #9):

*P13: "* *Data were collected using Microsoft Excel. The model was performed using C++ and statistical analyses were conducted using SAS software version 9.4. The threshold for statistical significance was a priori fixed at two-sided p<0.05. However, we prioritized effect sizes and confidence intervals over p-values, and presented R² and Pearson's R, as well as Mean Absolute Error (MAE) and Root Mean Square Error (RMSE) following current statistical practices [82,83]."*

## 11/ While R² and Pearson's R validate the model, overfitting or predictive accuracy is not explicitly assessed. Including cross-validation or out-of-sample testing would enhance validation robustness.

Answer: We appreciate the reviewer's insights regarding optimal validation approaches. Our model has minimal risk of overfitting since only one parameter required calibration while 93 parameters were derived directly from published literature and 1 was based on expert assumption. Nevertheless, we have strengthened our validation methodology by adding residual analysis to detect systematic errors, incorporating advanced metrics (MAE and RMSE) alongside correlation measures, and leveraging our out-of-sample validation against US data. We believe these approaches provide evidence of the model's predictive accuracy while acknowledging the computational constraints that make traditional k-fold cross-validation difficult for complex microsimulation models.

P10: "*Of the 95 model parameters, 93 were obtained from prior studies, one was based on expert judgment, while one parameter, the individual baseline risk of depression, could not be estimated from the literature and was therefore calibrated (S1 Table).*"

P12: "*Additionally, residual plots were generated to evaluate systematic biases and temporal patterns in prediction errors, and diagnostic plots were constructed to assess key model assumptions including independence of residuals over time.*"

P14: "*Residual analysis showed no systematic bias patterns, and diagnostic plots confirmed adequate model assumptions (temporal independence) (S5 Figure). Mean Absolute Error (MAE) and Root Mean Square Error (RMSE) were, respectively, 0.21 and 0.27 for SM use, and 0.005 and 0.006 for depression rates.*"

## 12/ Parameters like the 10% correlation coefficient between social media platforms seem arbitrary. Testing this assumption in sensitivity analyses would clarify its impact on results.

Answer: The 10% cross-platform correlation reflects an assumption grounded in empirical co-use patterns. We have now clarified this rationale and conducted sensitivity analyses showing minimal impact of this parameter on overall results (please see response to comment #9).

## 13/ The rationale for choosing specific parameter distributions (e.g., normal or uniform) is missing. Providing this information would clarify why these distributions were used for model inputs.

Answer: Our selection was guided by the statistical properties of each parameter: normal distributions for continuous variables with central tendencies (e.g., baseline risk), uniform distributions for bounded parameters without clear central values, and empirical distributions when available from literature. This rationale is now explained more clearly in the revised methods, as follows:

*P11: “Parameter distributions were selected based on the nature of the variable: normal distributions for continuous variables with central tendency (e.g., baseline risk), uniform for bounded parameters without clear central values, and empirical distributions when preserved from literature.”*

## Minor Comments

## 14/ Simulating 1,000 individuals per cohort may introduce sampling variability, especially for rare outcomes like suicide. Justification for this choice and its implications on uncertainty should be discussed.

Answer: Following this comment, we have added justification for the sample size choice and acknowledged its implications for rare outcomes.

*P11: “This sample size provides adequate statistical power for the primary outcome (depression prevalence) while maintaining computational feasibility. Although this may introduce sampling variability for rare outcomes such as suicide, our Monte Carlo approach with 500 iterations based on the random variation of all non-calibrated parameters simultaneously, either within a 95% confidence interval (CI) for parameters estimated from the literature or within a ±20% interval when CIs were not available, allows us to capture this uncertainty in the confidence intervals presented.”*

*P18: “Third, simulating 1,000 individuals per cohort may introduce sampling variability for rare outcomes like suicide.”*

## 15/ While parameter values are referenced in Supplementary Table 1, a concise summary of the most critical parameters and their uncertainty ranges should be included in the main text for clarity.

Answer: Following this comment, we have added a concise summary table of key parameters in the new Table 1.

## 16/ The integration of multiple data sources (e.g., INSEE, national surveys) is commendable, but handling of inconsistencies or missing data should be explicitly detailed.

Answer: We have added a short description of how inconsistencies or missing data were handled across sources.

*P13: “Data inconsistencies were resolved by prioritizing recent national surveys, and missing values were handled through linear interpolation or conservative assumptions within our ±20% uncertainty range.”*

## 17/ The "COVID-19 confinement" factor long-term effects on depression trends remain unclear. Discussing how this factor was validated or cross-checked would add robustness.

Answer: COVID-19 impact was modeled as a distinct risk factor for 2020, based on school closures. However, we agree with the reviewer that this parameter lacks long-term validation data, as the long-term effects of COVID-19 confinement on depression remain unclear. We now recognize this point in the limitation section.

*P18-19: “Sixth, the COVID-19 confinement factor was modeled based on observed 2020 data, but its long-term effects on depression trends remain uncertain and lack validation data.”*

## 18/ Scenarios like replacing 30 minutes of social media use with physical activity are idealistic. Addressing adherence and real-world feasibility would make these analyses more practical.

Answer: We agree with the reviewer and have added this point to the limitation section as follows:

*P19: “Ninth, the intervention scenarios modeled are hypothetical and not directly based on pilot studies or real-world trials. While some evidence supports individual components (e.g., physical activity benefits, social media time limits), we lack empirical data on the specific intervention designs tested here. This limits the practical applicability and real-world feasibility of our projections, as actual implementation may face unforeseen barriers, different adherence patterns, or unexpected outcomes not captured in our theoretical framework.”*

## 19/ The manuscript estimates societal costs but does not address the cost-effectiveness of proposed interventions. A dedicated analysis comparing intervention costs to benefits is recommended.

Answer: While we estimated in exploratory analyses the societal costs associated with depression reduction under different scenarios, we did not perform cost-effectiveness analyses comparing intervention costs to benefits, which is beyond the scope of our study. Following this comment, we acknowledge its importance and propose it as a future direction.

*P19: “Eleventh, while we estimated in exploratory analyses the societal costs associated with depression reduction under different scenarios, we did not perform cost-effectiveness analyses comparing intervention costs to benefits, which represents an important avenue for future research.”*

## 20/ The limitations section should explicitly address how unmeasured confounders, such as genetic predisposition, were mitigated or might bias results.

Answer: Unmeasured confounders (e.g., genetics) are now acknowledged more explicitly in the limitation section, as follows:

*P18: “First, as with all modeling studies, we rely on existing knowledge and current assumptions. Specifically, because depressive symptoms among adolescents result from a complex interaction of social, psychological, and biological factors, our model does not likely capture all contributing factors, and its results are potentially biased due to residual unmeasured confounding (e.g., genetic predisposition).”*

## 21/ Hypothetical interventions are assessed but not aligned with real-world pilot studies or trials. Incorporating data from similar initiatives would validate assumptions.

Answer: We now acknowledge this important limitation regarding the hypothetical nature of our intervention scenarios.

*P19: “Ninth, the intervention scenarios modeled are hypothetical and not directly based on pilot studies or real-world trials. While some evidence supports individual components (e.g., physical activity benefits, social media time limits), we lack empirical data on the specific intervention designs tested here. This limits the practical applicability and real-world feasibility of our projections, as actual implementation may face unforeseen barriers, different adherence patterns, or unexpected outcomes not captured in our theoretical framework. Tenth, the intervention scenarios modeled are hypothetical and not directly based on pilot studies or real-world trials, which may limit their practical applicability and adherence rates in actual implementation.”*

## 22/ Potential biases in data sources (e.g., underreporting of mental health issues or inaccuracies in self-reported social media usage) are not discussed and should be addressed.

Answer: Potential biases from self-reporting or underreporting are now discussed in the limitations.

*P19: “Seventh, although we primarily used data from national surveys with validated instruments when available, underreporting of mental health issues and inaccuracies in self-reported social media usage may have biased parameter estimates.”*

## 23/ The model assumes universal adherence to interventions, which is often unrealistic. Incorporating adherence rates from comparable interventions would make projections more realistic.

Answer: We agree with the Reviewer and now acknowledge this important limitation, as follows:

*P19: “Ninth, the intervention scenarios modeled are hypothetical and not directly based on pilot studies or real-world trials. While some evidence supports individual components (e.g., physical activity benefits, social media time limits), we lack empirical data on the specific intervention designs tested here. This limits the practical applicability and real-world feasibility of our projections, as actual implementation may face unforeseen barriers, different adherence patterns, or unexpected outcomes not captured in our theoretical framework. Tenth, the intervention scenarios modeled are hypothetical and not directly based on pilot studies or real-world trials, which may limit their practical applicability and adherence rates in actual implementation.”*

## 24/ Figures comparing observed vs. predicted prevalence rates lack detailed annotations (e.g., reasons for discrepancies in specific years). Enhanced visual clarity would aid interpretation. Figures such as Supplementary Figures 1 and 2 (adoption curves) lack explanation for assumptions about adoption trends. Clarifying whether these trends are universal or France-specific would be helpful.

Answer: Following this important comment, we have updated all Figures with clearer annotations and have expanded legends to explain discrepancies and assumptions.

## 25/ Supplementary Table 1 could group parameters by module (e.g., demographics, behaviours, outcomes) for easier interpretation. Including metadata, such as each parameter's role (e.g., input vs. calibrated), would improve usability.

Answer: Supplementary Table 1 is now reorganized by module and includes parameter’s roles. Please see S1 Table.

## 30/ Supplementary Table 2 details time spent on platforms but lacks longitudinal trends. Including these trends would clarify how usage patterns influence depression risks.

Answer: Longitudinal trends are now discussed with reference to the extrapolation method (please see S2 Table). We have also added the following note at end of this table: “*Peak usage periods were derived from modeled adoption curves (S1 and S2 Figures). Longitudinal trends were captured using gamma distribution extrapolation to model temporal variations influencing depression risk across birth cohorts.*”

## 31/ Sensitivity results for key parameters (e.g., baseline risk, COVID-19 impact) are not detailed. Including tornado diagrams or comprehensive outputs would improve transparency.

Answer: Following this comment, we have added sensitivity analyses, including tornado diagrams showing the individual impact of key parameters (baseline risk, COVID-19 impact, social media usage patterns) on model outputs, as requested. These sensitivity analyses demonstrate model robustness and are included in the supplementary material.

*P12: “We also performed three sensitivity analyses to assess model robustness: (1) tornado diagrams examining the individual impact of each key parameter on depression prevalence estimates, (2) testing alternative standard deviation values (10% and 30%, instead of 20%) for the gamma distribution used in social media usage extrapolations, and (3) testing different correlation coefficients (0.00 and 0.20, instead of 0.10) for cross-platform social media use. Results from all sensitivity analyses are reported with their impact on the primary outcome.”*

*P14: “Sensitivity analyses demonstrated model robustness across all tested parameters. Tornado diagrams (S7 Figure) showed that the baseline risk parameter had the largest influence on outcomes, while social media and risk factor parameters had moderate effects. Varying the standard deviation for social media usage distributions from 10% to 30% resulted in less than 5% variation in predicted depression prevalence (S8 Figure). Similarly, changing cross-platform correlation coefficients from 0% to 20% produced less than 5% variation in predicted depression prevalence (S9 Figure).”*

## 33/ While calibration is summarised (e.g., Supplementary Figure 5), the absence of residual plots or advanced goodness-of-fit metrics leaves gaps in assessing quality.

Answer: Following this comment, we have added comprehensive goodness-of-fit diagnostics and residual analysis to strengthen model validation.

*P12: “Additionally, residual plots were generated to evaluate systematic biases and temporal patterns in prediction errors, and diagnostic plots were constructed to assess key model assumptions including independence of residuals over time.”*

*P14: “Residual analysis showed no systematic bias patterns, and diagnostic plots confirmed adequate model assumptions (temporal independence) (S5 Figure). Mean Absolute Error (MAE) and Root Mean Square Error (RMSE) were, respectively, 0.21 and 0.27 for SM use, and 0.005 and 0.006 for depression rates.”*

## 34/ Figures use dotted lines for uncertainty ranges but could include shaded areas for better visual distinction.

Answer: Figures now include shaded areas instead of dotted lines for better visual distinction of uncertainty ranges.

## 35/ Some parameter estimates (e.g., for obesity and substance use) differ slightly between tables and figures. Consistency or explanations for discrepancies would avoid confusion.

Answer: All discrepancies in parameter values between tables and figures (initially due to use of different time periods) have been checked and resolved.

# Reviewer #2

## 36/ The authors provide a well-reasoned argument for the focus on social media and the challenges in disentangling its influence on rising adolescent depression rates. What is missing however is a clear explication of the gap this model is trying to fill. Why was individual-based microsimulation modelling used over other approaches e.g., agent-based modelling? Have studies previously used this approach to understand social media use (to my knowledge, they have not which highlights the novelty of this model)?

Answer: We thank the reviewer for this positive comment on our work. Following this comment, we tried to better articulate the methodological gap and novelty of our approach. We have enhanced the manuscript to clarify these points as follows:

*P5: “Individual-based microsimulation is a computational approach that models heterogeneous populations by simulating each person's life trajectory separately, incorporating their unique characteristics and risk factor profiles [29–33]. Unlike cohort-based models, this approach captures individual-level heterogeneity in risk factors, their accumulation over time, and differential responses to interventions. This method is particularly suited for evaluating complex policy interventions where effects vary across population subgroups. To our knowledge, this represents the first microsimulation model examining social media use and adolescent depression. We chose microsimulation over agent-based modeling because our research question focused on how individual-level risk factors (including social media exposure duration) accumulate over time to influence individual depression outcomes, rather than examining how behaviors or mental health states spread through social networks or how peer interactions influence individual outcomes.”*

## 37/ It is important also to provide some context for the choice of intervention scenarios being modelled. For example, one of the scenarios relates to time substitution from social media use to physical activity. Why was physical activity chosen? Is there any evidence to suggest that adolescents who engage in excessive social media use are less physically active? Is it not equally possible that time might be substituted from other activities e.g., sleeping which is also a risk factor for depressive symptoms? To be clear, the choice to look at physical activity is not an issue, I just think the paper would be strengthened by providing a better context around time substitution and evidence supporting the choice to look at physical activity over other activities.

Answer: We agree that the rationale for selecting physical activity as a substitution activity requires stronger justification. We have added evidence-based context to support this choice. We modified the manuscript accordingly, as follows:

*P10: “Physical activity and its threshold were chosen based on evidence from a recent systematic review and network meta-analysis showing that 30 minutes of exercise per session has significant beneficial effects on reducing depressive symptoms in adolescents [71]. Additionally, physical activity has well-established protective effects against depression [47] and evidence suggests inverse correlations between social media use and physical activity among adolescents [15,72,73]. While other activities could be considered, physical activity represents a clearly modifiable behavior with direct mental health benefits, and thus can be examined as a test case”*

## 38/ Could authors clarify if DALE - adjusted life expectancy loss - refers to Disability-adjusted life years (DALYs)? Or if authors are referring to Health-adjusted life expectancy (HALE)? DALE may confuse some readers as its not a term I've come across previously.

Answer: We thank the reviewer for this important clarification. DALE was indeed confusing terminology. We have corrected this throughout the manuscript to use the standard term HALE (Health-Adjusted Life Expectancy), including in the Table 2.

*P9: “We also projected the lifetime medical and societal costs linked to adolescent depressive symptoms and its care into adulthood, including costs related to depression recurrence, productivity loss, health-adjusted life expectancy loss (HALE), and suicide deaths and suicide attempts (Tables 1 and 2).”*

## 39/ The chosen interventions are interesting however, their effects are difficult to compare as they are all implemented over different time periods. Within the figures, it would be helpful to provide a line that shows the 'business-as-usual' or calibrated trajectory which shows the trends observed in the data, enabling readers to make a visual comparison of the potential impact of each scenario relative to the baseline case.

Answer: We agree that visual comparison would be enhanced by including the baseline trajectory. We have modified the supplementary figures 10 to 14 to include this reference line.

**40/ What was the timeframe for scenario 2 where social media use is limited to a maximum of 1 hour per day and scenario 4 where these limits were only imposed on high-risk adolescents? Were these over the same time period 2000-2022?**

Answer: The reviewer is right, scenarios 2 and 4 were implemented over the entire 2000-2022 period. We have clarified this in the methods and Table 2 footnotes.

*P9:* “*All intervention scenarios were evaluated against the current situation (i.e., observed social media use and depression trends in France between 2000-2022) over the complete study period to ensure comparable assessment.*”

*Table 2 footnotes: “^a^ Scenarios 2 and 4 (limiting social media use to 1 hour per day for all adolescents and for high-risk adolescents, respectively) were implemented over the entire 2000-2022 period.”*

## 41/ Why did authors decide to limit the timeframe of scenario 3 to 2016 and onwards? It makes it hard to compare which scenario results in the greatest reduction in the cumulative prevalence of depression when the timeframes are so different. This scenario seems almost as effective as scenario 2 where social media is limited to 1 hour max for all and makes me wonder whether it would have been more effective if it were implemented from 2000-2022 and not 2016-2022.

Answer: Thank you for identifying this lack of clarity in our original presentation. Upon review, we have standardized all intervention scenarios to the 2000-2022 period for fair comparison.

*P9-10: “We successively examined the following scenarios, for the period from 2000 to 2022: (i) The natural course of adolescent depression if no SM had been available; (ii) SM use would have been limited to a maximum of 1 hour per day. This threshold was conservatively chosen to be below the 2-hour threshold identified by Sala et al. as associated with negative mental health outcomes in their umbrella review [70]. This threshold was selected a priori to capture potentially beneficial effects of the intervention, especially in at-risk adolescents, while allowing adolescents to maintain some social media use for positive social connections and communication; and (iii) SM use would have been reduced by 30 minutes per day for those with a daily SM use of at least 1 hour per day and would have been replaced by 30 minutes per day of physical activity.”*

## 42/ A recent umbrella review by Sala focusing on the mental health impacts of social media use among adolescents found that > 2 hours of social media use per day was associated with negative mental health outcomes. It would also be helpful if authors could provide some justification or insight into why a 1 hour cut off for social media us was tested and others weren't considered.

Answer: We thank the reviewer for this comment. We selected the 1-hour threshold as a conservative intervention target for social media use limitations. The choice of this threshold was made to represent a precautionary approach below the 2-hour threshold identified by Sala et al. as associated with negative mental health outcomes in their umbrella review. However, we *a priori* chose this threshold to capture potentially beneficial effects of interventions on at-risk adolescents, while ensuring that adolescents could still benefit from positive aspects of social media use. We modified the manuscript as follows:

*P9-10: (ii) SM use would have been limited to a maximum of 1 hour per day. This threshold was conservatively chosen to be below the 2-hour threshold identified by Sala et al. as associated with negative mental health outcomes in their umbrella review [70]. This threshold was selected a priori to capture potentially beneficial effects of the intervention, especially in at-risk adolescents, while allowing adolescents to maintain some social media use for positive social connections and communication”*

## 43/ When authors mention 'the current situation' are they referring to the observed social media and depression trends or the scenario.

Answer: We have clarified that "the current situation" refers to the observed historical evolution of social media use and depression trends as simulated by our calibrated model.

*P9: “All intervention scenarios were evaluated against the current situation (i.e., observed social media use and depression trends in France between 2000-2022) over the complete study period to ensure comparable assessment.”*

***“****Table 2. Summary of projected effects of interventions applied between 2000 and 2022 on the cumulative lifetime prevalence of depression and its associated medical and social costs among the adolescents born between 1990 and 2012 in France (N=18.6 millions), as compared to the current situation (observed trends over the same 2000-2022 period).****”***

## 44/ On page 12, at the bottom of the results section authors state that "limiting social media use to 1 hour per day for all adolescents was associated [with] the greatest benefits in terms of medical and social costs…". This makes sense in relation to Table 1 which states that interventions all spanned 2000-2022. But per my earlier point, this seems to conflict with the methods section (p. 8) which states that "Starting in 2016 when average exposure to SM was greater than 90 min, SM use would have been reduced by 30 min per day …". Can authors please clarify the timeframes in both the text and table? If one intervention was only implemented from 2016 onwards, and the other from 2000, then I think it would be misleading to say that one is more effective than the other without explicitly qualifying this statement and the extent to which these different timeframes likely impacted the findings.

Answer: We thank the reviewer for this important comment. We have resolved this inconsistency by standardizing all intervention scenarios to the 2000-2022 period, ensuring fair comparison of effectiveness.

P10: “(iii) SM use would have been reduced by 30 minutes per day for those with a daily SM use of at least 1 hour per day and would have been replaced by 30 minutes per day of physical activity”

*P9: “All intervention scenarios were evaluated against the current situation (i.e., observed social media use and depression trends in France between 2000-2022) over the complete study period to ensure comparable assessment.”*

*P15-16: “Limiting social media use to 1 hour per day for all adolescents was associated with the greatest benefits in terms of medical and social costs associated with depression across the proposed measures, with all interventions modeled over the same study period (2000-2022) for direct comparison (Table 2).”*

*“Table 2. Summary of projected effects of interventions applied between 2000 and 2022 on the cumulative lifetime prevalence of depression and its associated medical and social costs among the adolescents born between 1990 and 2012 in France (N=18.6 millions), as compared to the current situation (observed trends over the same 2000-2022 period).”*

*Table 2 footnotes: “All interventions modeled over the same study period (2000-2022) for direct comparison."*

## 45/ Table 1, could authors clarify that scenario 2 where 30 minutes of social media use is replaced by 30 min of physical activity, that this replacement only occurs 'for those with daily use ≥ 1 hour per day.

Answer: We have added this important clarification as a footnote.

Footnotes Table 2: “ ^b^ The substitution of 30 minutes of SM use with 30 minutes of physical activity applied only to adolescents with daily SM use of ≥1 hour per day.”

## 46/ Could authors make a statement about the generalisability of the model's findings and the adaptability of the model to other contexts?

Answer: This is an important point. We have added a discussion of the model's generalizability and adaptability to other contexts.

*P17: "While this model was calibrated to French adolescent data and validated against US adolescent data, it demonstrates potential for broader applicability with limitations. The model framework could be adapted to other countries with appropriate parameter adjustments (demographics, behavior patterns, risk factor distributions). However, current representativeness is limited by data availability, as many countries lack comprehensive data on adolescent social media use and depression trends. Cultural differences in social media usage and mental health contexts may also influence model applicability."*

## 47/ Could authors explain in more detail how the curves in Appendix figure S2 were derived and the assumptions underpinning these?

Answer: Following this comment, we have enhanced the explanation of the adoption curve methodology.

*P6: “Social media platforms, comprising Facebook, Twitter, WhatsApp, Snapchat, TikTok, and Instagram, were included in the model to closely mirror real-world usage [37]. Each platform was attributed a probability of use for each individual aged 10 to 17 years, influenced by sex, age, and the year of introduction of each social medium. The adoption curves for each social media platform were modeled using the Bass diffusion framework applied to actual historical uptake data from Mediamétrie [37], with platform-specific timing based on market entry years and typical adolescent saturation dynamics. A typical adoption curve [38] for use of social media was modeled according to actual utilization data [37], starting with Facebook's introduction in 2004. The shape of the curve is described in supplementary material (S1 Figure and S2 Figure). Daily usage durations per platform were estimated based on specific data from France [37] and were extrapolated using a gamma distribution, which appropriately captures the right-skewed nature of social media usage where most users have moderate usage while some have very high usage [39], assuming a coefficient of variation (SD/mean) of 20%. The gamma distribution parameters were applied uniformly across platforms as a simplifying assumption, even if platform-specific distributions may exist.”*

*S2 Figure Footnote: “Note: The curves are derived from the Bass diffusion model applied to historical data from Mediamétrie, accounting for each platform's market entry year (Facebook 2004, Twitter 2006, WhatsApp 2009, Instagram 2010, Snapchat 2011, TikTok 2016) and typical adolescent adoption patterns. The S-shaped curves reflect initial slow adoption, rapid growth phase, and eventual market saturation dynamics observed in real-world social media uptake among French adolescents.”*

## Minor comments

## 48/ On page 9, under section 2.7, "…general population exposed to SN" and page 10, "…adolescents exposed to SN between…" - please change to SM. More generally please define social media as SM and use the acronym consistently in all subsequent mentions.

Answer: Following this comment, we have corrected all mentions from “SN” to “SM” and ensured consistent use of this acronym throughout the manuscript.

*P11: “Results were extrapolated to the French general population exposed to SM, i.e. aged 10 to 17 years between 1990 et 2012, based on an average of 790,000 birth per year between 1990 and 2012.”*

*P12: “Finally, we compared the outcomes of the different interventions to the current situation for the main outcome and projections for adolescent exposed to SM between 2004 and 2022.”*

## 49/ On page 12, at the bottom of the results section authors state that "limiting social media use to 1 hour per day for all adolescents was associated [with] the greatest benefits in terms of medical and social costs…". I think there is a 'with' missing.

Answer: Thank you for noticing this missing word. We have now added it.

*P15-16: “Limiting social media use to 1 hour per day for all adolescents was associated with the greatest benefits in terms of medical and social costs associated with depression across the proposed measures, with all interventions modeled over the same study period (2000-2022) for direct comparison (Table 2).”*

# Reviewer #3

## 50/ I believe the background literature and rationale are mostly sufficient. However, I believe there are some more recent studies that could be used in some sections to enhance the robustness of the assumptions. There are some very recent papers outlining links (or lack of evidence of links) between SM use and psychological distress. The recent literature suggests that it is not as simple as more time on SM = increases in mental health problems. The relationship is complex and affected by several factors such as engagement with SM (i.e. active versus passive engagement), experiences with cyberbullying, who and how young people are interacting with (i.e. friend or foe) and their own engagement in terms of posting. This needs to be accounted for or considered in more detail at least in the intro and discussion, and perhaps in the model if at all possible.

Answer: We thank the reviewer for this important point. Following it, we have substantially expanded both the introduction and discussion to acknowledge the complexity of the SM-depression relationship, including factors such as active vs passive use, cyberbullying, and social context. While our model focuses on usage duration as a primary exposure metric, we now explicitly discuss these nuances, and their implications as follows:

*P4: “Finally, recent evidence suggests the relationship between social media use and adolescent mental health could be more nuanced than simple dose-response effects. Factors such as the type of engagement (active posting versus passive scrolling), exposure to cyberbullying, the nature of online interactions (supportive versus hostile), and individual differences in vulnerability may all moderate this relationship [21,22].”*

*P19: “Twelfth, our model captures social media exposure through duration of use, which represents only one dimension of a complex phenomenon. We did not model qualitative aspects such as active versus passive use, content type, or social context—factors that prior research suggests significantly moderate mental health impacts [21,22,26,94,95]. Future studies would benefit in incorporating these data once they will become available.”*

## 51/ In section 2 (Methods) I would like to see a few sentences to summarize the method rather than just referring to previous studies. Currently it says "Following previously described methods [27-31]".

Answer: Following this comment, we have added a brief description of microsimulation modeling, as follows:

*P5: “Individual-based microsimulation is a computational approach that models heterogeneous populations by simulating each person's life trajectory separately, incorporating their unique characteristics and risk factor profiles [29–33]. Unlike cohort-based models, this approach captures individual-level heterogeneity in risk factors, their accumulation over time, and differential responses to interventions. This method is particularly suited for evaluating complex policy interventions where effects vary across population subgroups. To our knowledge, this represents the first microsimulation model examining social media use and adolescent depression. We chose microsimulation over agent-based modeling because our research question focused on how individual-level risk factors (including social media exposure duration) accumulate over time to influence individual depression outcomes, rather than examining how behaviors or mental health states spread through social networks or how peer interactions influence individual outcomes. Following previously described methods [29–33], we built an individual-based microsimulation model to test the hypothesis that social media (SM) use increases the risk of depression among adolescents.”*

## 52/ The model description and justification are clear and detailed, and the purpose and scope are evident and justified. It appears that the data used in the model are appropriate to the context and population under study, and have been drawn from recent statistics or (mostly) recent studies - based on my earlier comment there may perhaps be some that could be more updated. Parameters seem to be appropriately selected. Validation and calibration are well reported. Can the authors expand on any sensitivity analysis carried out in terms of if any assumptions or parameters were changed did this affect the model outcomes?

Answer: We thank the reviewer for the positive assessment. We have now conducted and reported three sensitivity analyses that demonstrate model robustness across key assumptions, as follows:

*P 12: “Additionally, residual plots were generated to evaluate systematic biases and temporal patterns in prediction errors, and diagnostic plots were constructed to assess key model assumptions including independence of residuals over time. We also performed three sensitivity analyses to assess model robustness: (1) tornado diagrams examining the individual impact of each key parameter on depression prevalence estimates, (2) testing alternative standard deviation values (10% and 30%, instead of 20%) for the gamma distribution used in social media usage extrapolations, and (3) testing different correlation coefficients (0.00 and 0.20, instead of 0.10) for cross-platform social media use. Results from all sensitivity analyses are reported with their impact on the primary outcome.”*

*P14: “Sensitivity analyses demonstrated model robustness across all tested parameters. Tornado diagrams (S7 Figure) showed that the baseline risk parameter had the largest influence on outcomes, while social media and risk factor parameters had moderate effects. Varying the standard deviation for social media usage distributions from 10% to 30% resulted in less than 5% variation in predicted depression prevalence (S8 Figure). Similarly, changing cross-platform correlation coefficients from 0% to 20% produced less than 5% variation in predicted depression prevalence (S9 Figure).”*

# Comments from the academic editor:

## 53/ Please ensure that the causality in the model is sufficiently justified and discuss the evidence on which it is based, e.g. small prospective studies/trials where the key question would be the representativeness and transferability of the estimated effect on depression/outcomes of however they changed media consumption, or observational studies documenting relationships that are either cross-sectional or prospective.

Answer: We thank the Editor for this important comment on causality justification. Our model integrates multiple lines of evidence, while acknowledging the challenges of establishing definitive causal relationships in this field.

Our approach draws on experimental evidence from randomized controlled trials (Introduction, p4): "Experimental evidence also suggests that curbing social media use may bolster mental health well-being among adolescents [18-20]," citing Hunt et al. (2018), Lambert et al. (2022), and Faulhaber et al. (2023). While these trials demonstrate beneficial effects of limiting social media use, we recognize their relatively small sample sizes and short durations.

Additionally, we incorporate findings from Liu et al.'s meta-analysis (2022) [ref 17] of longitudinal studies, which found a modest but significant association (OR = 1.13, 95%CI: 1.09-1.17) between SM usage and adolescent depression. As we state in our Methods (p5): "establishing a potential causal relationship [28] between social media use and adolescent depression is challenging" due to practical constraints and multiple confounders.

Our model's validation against US data provides support for the transferability of these relationships across different populations, though we acknowledge this area requires continued research.

## 54/ Please ensure that you have adequately adjusted for confounding and discussed potential unaddressed confounding in detail. Have you considered internet use as a coping mechanism?

Answer: Our model addresses confounding by incorporating eight major risk factors based on established literature (Methods, p7-8): sex, age, parental psychiatric history, childhood adversities, physical inactivity, obesity/overweight, chronic physical diseases, and substance use.

We explicitly acknowledge limitations in our Discussion (p18): "our model does not likely capture all contributing factors, and its results are potentially biased due to residual unmeasured confounding (e.g., genetic predisposition)."

Regarding social media as a coping mechanism, we recognize this represents an important consideration. While our model structure assumes social media use precedes depression, we acknowledge that reverse causality may exist . The validation of our model against real-world depression trends suggests it captures important patterns, though bidirectional relationships warrant future investigation.

**55/ Please discuss the generalizability/transferability of the effect size.**

Answer: We discuss generalizability with appropriate nuance in our Discussion (p17): "While this model was calibrated to French adolescent data and validated against US adolescent data, it demonstrates potential for broader applicability with limitations. The model framework could be adapted to other countries with appropriate parameter adjustments."

We further specify (p19-20): "Regional variations in mental health care access, cultural attitudes toward mental health, family structures, and social media usage patterns may limit the generalizability of these assumptions." However, we note that "the adequate validation against US data suggests that the core relationships modeled may be sufficiently robust to capture depression trends across different healthcare and social systems, at least for high-income countries with similar social media penetration."

## 56/ Please ensure that the above points are addressed and documented in detail in your point-by-point response and in the manuscript.

Answer: All points raised by the Academic Editor and reviewers have been thoroughly addressed in this point-by-point response and through corresponding revisions in the manuscript (highlighted in yellow).

We warmly thank the academic editor and the reviewers for their important help in improving our manuscript.

# General editorial requests:

*(Note: not all will apply to your paper, but please check each item carefully)*

## 57/ We ask every co-author listed on the manuscript to fill in a contributing author statement, making sure to declare all competing interests. If any of the co-authors have not filled in the statement, we will remind them to do so when the paper is revised. If all statements are not completed in a timely fashion this could hold up the re-review process. If new competing interests are declared later in the revision process, this may also hold up the submission. Should there be a problem getting one of your co-authors to fill in a statement we will be in contact. Please do not add or remove authors without first discussing this with the handling editor. You can see our competing interests policy here: <http://journals.plos.org/plosmedicine/s/competing-interests>.

Answer: None of the authors have any competing interests to disclose. This is stated in the Competing Interests section of the manuscript.

## 58/ Please upload any figures associated with your paper as individual TIF or EPS files with 300dpi resolution at resubmission; please read our figure guidelines for more information on our requirements: <http://journals.plos.org/plosmedicine/s/figures>. While revising your submission, please upload your figure files to the PACE digital diagnostic tool, <https://pacev2.apexcovantage.com/>. PACE helps ensure that figures meet PLOS requirements. To use PACE, you must first register as a user. Then, login and navigate to the UPLOAD tab, where you will find detailed instructions on how to use the tool. If you encounter any issues or have any questions when using PACE, please email us at [PLOSMedicine@plos.org](mailto:PLOSMedicine@plos.org).

Answer: All figures are uploaded as high-resolution editable files.

## 59/ Please ensure that the paper adheres to the PLOS Data Availability Policy (see <http://journals.plos.org/plosmedicine/s/data-availability>), which requires that all data underlying the study's findings be provided in a repository or as Supporting Information. For data residing with a third party, authors are required to provide instructions with contact information (web or email address) for obtaining the data. Please note that a study author cannot be the contact person for the data. PLOS journals do not allow statements supported by "data not shown" or "unpublished results." For such statements, authors must provide supporting data or cite public sources that include it.

Answer: All data and code used throughout this manuscript is shared in the public repository https://github.com/henrileleu/MicrosimDepressionSM. This is also stated in section “2.9. Code availability” of the manuscript.

## 60/ We expect all researchers with submissions to PLOS in which author-generated code underpins the findings in the manuscript to make all author-generated code available without restrictions upon publication of the work. In cases where code is central to the manuscript, we may require the code to be made available as a condition of publication. Authors are responsible for ensuring that the code is reusable and well documented. Please make any custom code available, either as part of your data deposition or as a supplementary file. Please add a sentence to your data availability statement regarding any code used in the study, e.g. "The code used in the analysis is available from Github [URL] and archived in Zenodo [DOI link]" Please review our guidelines at <https://journals.plos.org/plosmedicine/s/materials-software-and-code-sharing> and ensure that your code is shared in a way that follows best practice and facilitates reproducibility and reuse. Because Github depositions can be readily changed or deleted, we encourage you to make a permanent DOI'd copy (e.g. in Zenodo) and provide the URL.

Answer: All data and code used throughout this manuscript is shared in the public repository https://github.com/henrileleu/MicrosimDepressionSM. This is also stated in section “2.9. Code availability” of the manuscript.

## 61/ FINANCIAL DISCLOSURES: The funding statement should include: specific grant numbers, initials of authors who received each award, URLs to sponsors’ websites. Also, please state whether any sponsors or funders (other than the named authors) played any role in study design, data collection and analysis, the decision to publish, or preparation of the manuscript. If they had no role in the research, include this sentence: “The funders had no role in study design, data collection and analysis, decision to publish, or preparation of the manuscript.”

Answer: This work had no funding or financial support.

## 62/ COMPETING INTEREST: All authors must declare their relevant competing interests per the PLOS policy, which can be seen here: <https://journals.plos.org/plosmedicine/s/competing-interests>. For authors with ties to industry, please indicate whether any of the interests has a financial stake in the results of the current study.

Answer: None of the authors have any competing interests to disclose. This is stated in the Competing Interests section of the manuscript.

## 63/ DATA AVAILABILITY: The Data Availability Statement (DAS) requires revision. For each data source used in your study: a) If the data are freely or publicly available, note this and state the location of the data: within the paper, in Supporting Information files, or in a public repository (include the DOI or accession number). b) If the data are owned by a third party but freely available upon request, please note this and state the owner of the data set and contact information for data requests (web or email address). Note that a study author cannot be the contact person for the data. c) If the data are not freely available, please describe briefly the ethical, legal, or contractual restriction that prevents you from sharing it. Please also include an appropriate contact (web or email address) for inquiries (again, this cannot be a study author).

Answer: All data and code used throughout this manuscript is shared in the public repository https://github.com/henrileleu/MicrosimDepressionSM. This is also stated in section “2.9. Code availability” of the manuscript.

## 64/ ETHICS STATEMENT: Please include a short statement on why your study did not require ethical approval.

Answer: This study did not require ethical approval as it relied exclusively on publicly available aggregate data from national surveys and administrative databases, with no direct collection of individual participant data or human subjects involvement. This is now stated in the methods, section “2.10. Ethics statement”.

# FORMATTING – GENERAL

## 65/ Abstract: Please structure your abstract using the PLOS Medicine headings (Background, Methods and Findings, Conclusions). Please combine the Methods and Findings sections into one section.

Answer: Done.

## 66/ At this stage, we ask that you include a short, non-technical Author Summary of your research to make findings accessible to a wide audience that includes both scientists and non-scientists. The Author Summary should immediately follow the Abstract in your revised manuscript. This text is subject to editorial change and should be distinct from the scientific abstract. Ideally each sub-heading should contain 2-3 single sentence, concise bullet points containing the most salient points from your study. In the final bullet point of 'What Do These Findings Mean?', please include the main limitations of the study in non-technical language. Please see our author guidelines for more information: <https://journals.plos.org/plosmedicine/s/revising-your-manuscript#loc-author-summary>.

Answer: Done.

## 67/ Please express the main results with 95% CIs as well as p values. When reporting p values please report as p<0.001 and where higher as the exact p value p=0.002, for example. Throughout, suggest reporting statistical information as follows to improve clarity for the reader "22% (95% CI [13%,28%]; p</=)". Please be sure to define all numerical values at first use.

Answer: Done.

## 68/ Please include page numbers and line numbers in the manuscript file. Use continuous line numbers (do not restart the numbering on each page).

Answer: Done.

## 69/ Please cite the reference numbers in square brackets. Citations should precede punctuation.

Answer: Done.

## 70/ FIGURES AND TABLES

## Please provide titles and legends for all figures and tables (including those in Supporting Information files).

## Please define all abbreviations used in each figure/table (including those in Supporting Information files).

## Please consider avoiding the use of red and green in order to make your figure more accessible to those with color blindness.

Answer: Done.

## 71/ SUPPLEMENTARY MATERIAL

## Please note that supplementary material will be posted as supplied by the authors. Therefore, please amend it according to the relevant comments outlined here.

## Please cite your Supporting Information as outlined here: <https://journals.plos.org/plosmedicine/s/supporting-information>

Answer: Done.

## 72/ REFERENCES

## PLOS uses the numbered citation (citation-sequence) method and first six authors, et al.

## Please ensure that journal name abbreviations match those found in the National Center for Biotechnology Information (NCBI) databases (<http://www.ncbi.nlm.nih.gov/nlmcatalog/journals>), and are appropriately formatted and capitalised.

## Where website addresses are cited, please include the complete URL and specify the date of access (e.g. [accessed: 12/06/2023]).

## Please also see <https://journals.plos.org/plosmedicine/s/submission-g>uidelines#loc-references for further details on reference formatting.

Answer: Done.

# STUDY TYPE-SPECIFIC REQUESTS

*The following list is derived from Geoffrey P Garnett, Simon Cousens, Timothy B Hallett, Richard Steketee, Neff Walker. Mathematical models in the evaluation of health programmes. (2011) Lancet DOI:10.1016/S0140-6736(10)61505-X:*

## 73/ If pertinent, please provide a diagram that shows the model structure, including how the natural history of the disease is represented, the process and determinants of disease acquisition, and how the putative intervention could affect the system.

Answer: The model structure, natural history representation, disease acquisition determinants, and intervention effects are described in the Methods section.

## 74/ Please provide a complete list of model parameters, including clear and precise descriptions of the meaning of each parameter, together with the values or ranges for each, with justification or the primary source cited and important caveats about the use of these values noted.

Answer: Model parameters are fully described in Table 1 and S1 Table.

## 75/ Please provide a clear statement about how the model was fitted to the data, including goodness-of-fit measure, the numerical algorithm used, which parameter varied, constraints imposed on parameter values, and starting conditions.

Answer: All information regarding the fit of the data, goodness-of-fit, algorithm description, parameters, starting conditions and constrains is fully described in the methods section.

## 76/ For uncertainty analyses, please state the sources of uncertainties quantified and not quantified [can include parameter, data, and model structure].

Answer: All sources of uncertainties are detailed in the methods, section “2.7. Statistical analysis”.

## 77/ Please provide sensitivity analyses to identify which parameter values are most important in the model. Uncertainty estimates seek to derive a range of credible results on the basis of an exploration of the range of reasonable parameter values. The choice of method should be presented and justified.

Answer: All sensitivity analyses are described in the methods and results sections.

## 78/ Please discuss the scientific rationale for the choice of model structure and identify points where this choice could influence conclusions drawn. Please also describe the strength of the scientific basis underlying the key model assumptions.

Answer : Scientific rationale and strength of the scientific basis are described in the Methods and Discussion sections.

## 79/ For studies that develop a prediction model or evaluate its performance, please ensure that the study is reported according to the TRIPOD statement (<https://www.equator-network.org/reporting-guidelines/tripod-statement>) and include the completed checklist as Supporting Information. Please add the following statement, or similar, to the Methods: "This study is reported as per the Transparent Reporting of a Multivariable Prediction Model for Individual Prognosis Or Diagnosis (TRIPOD) statement (S1 Checklist)." For studies using machine learning, please use the TRIPOD-AI checklist. When completing the checklist, please use section and paragraph numbers, rather than page numbers.

Answer: The TRIPOD-AI checklist is attached as the supplementary material S1 Checklist.
