# Supplementary material for: Impact of excessive social media use on adolescent depression and its consequences in France: An individual-based microsimulation model
Source: PLoS Med. 2025 Oct 21;22(10):e1004737. doi: 10.1371/journal.pmed.1004737 (PMC12539716; doi:10.1371/journal.pmed.1004737)
Supplement: Attachment — Submitted filename: response_to_reviewers_auresp_3.docx [file pmed.1004737.s019.docx]

August 22th, 2025,

Dear Editor,

Thank you very much for giving us the opportunity to submit a revised version of our manuscript PMEDICINE-D-24-03990R2 “Impact of Excessive Social Media Use On Adolescent Depression and Its Consequences in France: An Individual-Based Microsimulation Model” to be considered for publication in PLOS Medicine.

We appreciate the time and effort that you and the reviewers have dedicated in providing valuable feedback on our manuscript. A point-by-point response is detailed below, and we have highlighted the corresponding changes in yellow within the manuscript.

The manuscript contains no data, patient information, or other material or results that have been published elsewhere. It has not been submitted to any other journal, nor have the results been presented at any scientific meeting.

We thank you in advance for considering this contribution.

Best regards,

Nicolas HOERTEL, MD, MPH, Ph.D.,

On behalf of all co-authors,

Université Paris Cité, AP-HP Centre, DMU Psychiatrie et Addictologie,

Corentin Celton Hospital, Issy-les-Moulineaux, France

Comments from Reviewers

## Reviewer #1: Thank you for thoroughly addressing all my comments and providing detailed clarifications in your revised manuscript. I appreciate the effort you have put into enhancing the rigor and transparency of your analyses. The manuscript now reflects a robust and valuable contribution to subject, I am happy to recommend this work for publication and look forward to seeing its positive impact in the field.

## Reviewer #2: The authors have done a great job revising the manuscript and have thoroughly addressed all the points from my review. This is a strong piece of work that will make a valuable contribution to the field. Congratulations to the authors.

## Reviewer #3: Thank you to the authors for thoroughly attending to all the reviewer comments in such detail. This has further improved the quality and transparency of the study. Well done.

Answer: We sincerely thank the reviewers for their very encouraging feedback. Their constructive input throughout the review process has greatly strengthened our work, and we are delighted they found our revisions satisfactory.

Requests from Editors

## 1/ Please confirm that your title complies with to PLOS Medicine's style. Your title must be nondeclarative and not a question. It should begin with main concept if possible. "Effect of" should be used only if causality can be inferred, i.e., for an RCT. Please place the study design ("A randomized controlled trial," "A retrospective study," "A modelling study," etc.) in the subtitle (i.e., after a colon).

Answer: We modified the title in order to comply with PLOS Medicine’s style, as follows: “*Impact of Excessive Social Media Use On Adolescent Depression and Its Consequences in France: An Individual-Based Microsimulation Model*”.

## 2/ Statistical reporting: Please revise throughout the manuscript, including tables and figures.

- **Please report statistical information as follows to improve clarity for the reader ""22% (95% CI [13,28]; p</=)"".**
- **Please separate upper and lower bounds with commas instead of hyphens as the latter can be confused with reporting of negative values.**
- **Please repeat statistical definitions (HR, CI etc.) for each set of parentheses.**

Answer: All statistical reporting has been revised throughout the manuscript as requested.

## 3/ Please ensure that all abbreviations are defined at first use throughout the text (including statistical abbreviations).

Answer: All abbreviations have been defined at first use throughout the text.

## 4/ Please ensure that tables and figures, including those in supplementary files, are appropriately referenced in the main text.

Answer: All tables and figures are now appropriately referenced in the main text.

## 5/ Please review your text for claims of novelty or primacy (e.g. 'for the first time' or ‘novel’) and remove this language.

Answer: The manuscript was modified accordingly.

## 6/ Please check that any use of statistical terms (such as trend or significant) are supported by the data, and if not please remove them. The term trend should be used only when the test for trend has been conducted.

Answer: Done.

## 7/ Please provide titles and legends for all figures and tables (including those in Supporting Information files). Please define all acronyms used in each figure or table in its corresponding legend.

Answer: Done.

## 8/ Please remove the numbering from the headings.

Answer: Done.

## 9/ Please confirm that the information you provided in the online submission form is up to date and accurate.

Answer: We confirm that all provided information in the online submission is up to date and accurate.

# ABSTRACT

## 10/ Please confirm that your abstract complies with our requirements, including providing all the information relevant to this study type https://journals.plos.org/plosmedicine/s/submission-guidelines#loc-abstract

Answer: Confirmed.

## 11/ Abstract Background: The final sentence should clearly state the study question.

Answer: We modified the abstract as follows: “This study examined whether excessive SM use contributes to rising adolescent depression rates and evaluated potential mitigation strategies.”

## 12/ Please include the study design, population and setting, number of participants, years during which the study took place, length of follow up, and main outcome measures.

Answer: We have included all required elements in the Methods and Findings section, as follows:

*Methods and Findings: “We developed an individual-based microsimulation model of 18.6 million French adolescents born 1990-2012, tracking depression outcomes from 2000-2022 (analyses conducted August 2024-July 2025). The model incorporated 95 parameters including demographics, SM use patterns, and established depression risk factors (childhood adversities, chronic physical conditions, physical inactivity, obesity, substance use). The main outcome was cumulative depression cases and secondary outcomes were suicide deaths, health-adjusted life expectancy (HALE) loss, and associated costs. The model was well-calibrated and validated adequately against US-specific data.”*

## 13/ Please quantify the main results (with 95% CIs and p values).

Answer: We quantified main results with 95% CIs but not p-values, as p-values are not meaningful in microsimulation models where statistical significance depends directly on the number of simulation iterations rather than sampling variability. With sufficient iterations, any difference becomes 'significant' regardless of practical importance. Instead, we followed best practices for simulation studies by reporting effect sizes, credible intervals, and model performance metrics (R², Pearson's R, MAE, RMSE) which better capture model accuracy and uncertainty, in line with the recommendations done by the reviewers. We modified the abstract as follows:

*Methods and Findings: “(…) Among French adolescents, simulations indicated that excessive SM use was associated with an additional cumulative lifetime 590,000 depression cases (95%CI [400,000, 760,000]), 799 suicide deaths (95%CI [547, 1,028]), 137,000 (95%CI [94,000, 176,000]) HALE loss years, and 3.94 (95%CI [2.70, 5.07]) billion euros, compared to scenarios without SM platforms.”*

## 14/ Please include the important dependent variables that are adjusted for in the analyses (if too many, please sure overarching categories).

Answer: We modified the abstract as follows:

*Methods and Findings: “The model incorporated 95 parameters including demographics, SM use patterns, and established depression risk factors (childhood adversities, chronic physical conditions, physical inactivity, obesity, substance use).”*

## 15/ Please ensure that all numbers presented in the abstract are present and identical to numbers presented in the main manuscript text.

Answer: We verified that all numerical values in the revised abstract are identical to those presented in the main manuscript.

## 16/ In the last sentence of the Abstract Methods and Findings section, please describe the main limitation(s) of the study's methodology.

Answer: We added the following sentence: “*Key limitations are that microsimulation modeling cannot establish causality from observational data and the reliance on duration-based exposure measures without capturing content type or engagement quality”.*

**17/ Abstract Conclusions: Please address the study implications without overreaching what can be concluded from the data; the phrase "In this study, we observed ..." may be useful. Please interpret the study based on the results presented in the abstract, emphasizing what is new without overstating your conclusions.**

Answer: We revised the Abstract as follows:

*“Conclusions: In this study, we estimated that limiting SM use to 1 hour per day for all adolescents, replacing 30 minutes of SM use with 30 minutes of physical activity, or stopping its use for adolescents most at-risk for depression, would be associated with a reduction in cumulative lifetime prevalence of depression by 14.7%, 12.9%, and 12.0%, respectively, and diminished associated costs. Targeted social media interventions could potentially reduce adolescent depression burden, though real-world implementation and effectiveness require validation.”*

# AUTHOR SUMMARY

## 18/ Under ‘What Did the Researchers Do and Find?’, we suggest adding an additional bullet point that briefly explains what the aim of the microsimulation model was.

Answer: We added the following bullet point: “*The model aimed to test whether excessive social media use contributes to rising adolescent depression rates and to evaluate mitigation strategies*.”

## 19/ In the final bullet point of 'What Do These Findings Mean?', please include the main limitations of the study in non-technical language.

Answer: We included the final bullet points: *“Real-world implementation and effectiveness of these interventions require validation”* and *“Limitations include inability to determine causality and potential under reporting of SM use.”*

# INTRODUCTION

## 20/ Please ensure that the Introduction ends with a clear description of the study question or hypothesis.

Answer: We added a clear statement of the study question at the end of the Introduction section.

*L133-135: “In this study, we hypothesized that excessive social media use substantially contributes to rising adolescent depression rates, and that targeted social media interventions could potentially reduce adolescent depression burden.”*

# METHODS AND RESULTS

## 21/ Please add the following statement, or similar, to the Methods: "This study is reported as per the Transparent Reporting of a Multivariable Prediction Model for Individual Prognosis Or Diagnosis (TRIPOD-AI) statement (S1 Checklist)." When completing the checklist, please use section and paragraph numbers, rather than page numbers.

Answer: This statement has been added to the Methods section (2.7. Statistical Analysis), and the TRIPOD-AI checklist (S1 Checklist) has been completed using section and paragraph numbers as requested and included in the supplementary material.

## 22/ When reporting age, please add a unit, such as years.

Answer: Done.

## 23/ l.292: Please note that this information (Data availability and Code availability) should be included in the Data Availability Statement in the online submission form. You may remove the links from the main text and simply describe the data sources and how they were used.

Answer: The data availability section in the manuscript was modified to remove all links from the main text. Access to the data sources is now only referenced. All necessary information is included in the Data Availability Statement.

## 24/ Figure 1: Please define the meaning of the orange triangles in the figure legend. Please note that the figure description appears to repeat certain information. Please revise.

Answer: We have updated Figure 1 legend to define both orange squares ('Observed USA (12-17 years)') and blue triangles ('Observed France (15-24 years)'). The figure description has been revised to eliminate redundancies.

## 25/ l.356ff, “Limiting social media use to 1 hour per day for all adolescents was associated with the greatest benefits in terms of medical and social costs…” – we suggest describing the cost results in more detail here.

Answer: This sentence was modified in order to detail the cost results, as follows:

*L376-379: “Limiting social media use to 1 hour per day for all adolescents was associated with the greatest benefits in terms of medical and social costs associated with depression across the proposed measures, generating total projected cost savings of 2.20 billion euros (95%CI [0.63, 3.56]), with all interventions modeled over the same study period (2000-2022) for direct comparison (Table 2).”*

## 26/ Please specify the variables controlled for in all relevant Tables.

Answer: Table 2 footnotes were updated to explicitly list all variables controlled for in the analyses.

*“Note 2: All analyses controlled for sex, age, parental history of psychiatric disorders, childhood adversities (physical abuse, emotional abuse, sexual abuse, neglect, bullying), physical inactivity, obesity and overweight, chronic physical conditions, substance use (alcohol, tobacco, cannabis), and COVID-19 pandemic effects. Model parameters are detailed in S1 Table.”*

## 27/ We find the findings in Figures S10 and S11 very interesting and useful. They might be worth moving from the supplementary information to the main text. However, we will leave this decision to you.

Answer: We thank the Editor for this suggestion. We have moved Figures S10 and S11 from the supplementary material to the main text as Figure 4 and Figure 5, respectively. The numbering of all remaining supplementary figures has been adjusted accordingly to maintain sequential order.

General Editorial Requests

## 28/ (1) We ask every co-author listed on the manuscript to fill in a contributing author statement. If any of the co-authors have not filled in the statement, we will remind them to do so when the paper is revised. If all statements are not completed in a timely fashion this could hold up the re-review process. Should there be a problem getting one of your co-authors to fill in a statement we will be in contact. YOU MUST NOT ADD OR REMOVE AUTHORS UNLESS YOU HAVE ALERTED THE EDITOR HANDLING THE MANUSCRIPT TO THE CHANGE AND THEY SPECIFICALLY HAVE AGREED TO IT.

Answer: The Author Contributions section of the manuscript has been updated in accordance with CRediT Taxonomy guidelines. However, we were unable to find a specific contributing author statement form online to be signed by each author.

## 29/ (2) Please ensure that the paper adheres to the PLOS Data Availability Policy (see http://journals.plos.org/plosmedicine/s/data-availability), which requires that all data underlying the study's findings be provided in a repository or as Supporting Information. For data residing with a third party, authors are required to provide instructions with contact information for obtaining the data. PLOS journals do not allow statements supported by "data not shown" or "unpublished results." For such statements, authors must provide supporting data or cite public sources that include it.

Answer: All code and data underlying the study’s findings can be found in a public GitHub repository, as stated in Code Availability (<https://github.com/henrileleu/MicrosimDepressionSM>). All third-party data is included and referenced in the main text.

## 30/ (3) Please review your reference list to ensure that it is complete and correct. If you have cited papers that have been retracted, please include the rationale for doing so in the manuscript text, or remove these references and replace them with relevant current references. Any changes to the reference list should be mentioned in the rebuttal letter that accompanies your revised manuscript.

Answer: All references were thoroughly reviewed to ensure completeness and correctness. References 81 to 86 were added in this new version to clearly cite third-party data sources used in this study (comment #29), while removing all direct links from the main text as requested (comment #23).
